# SPP1^+^ TAM Regulates the Metastatic Colonization of CXCR4^+^ Metastasis‐Associated Tumor Cells by Remodeling the Lymph Node Microenvironment

**DOI:** 10.1002/advs.202400524

**Published:** 2024-09-05

**Authors:** Liang Dong, Shujun Hu, Xin Li, Shiyao Pei, Liping Jin, Lining Zhang, Xiang Chen, Anjie Min, Mingzhu Yin

**Affiliations:** ^1^ Department of Dermatology Hunan Engineering Research Center of Skin Health and Disease Hunan Key Laboratory of Skin Cancer and Psoriasis Xiangya Hospital Central South University Changsha Hunan 410008 China; ^2^ Clinical Research Center (CRC) Medical Pathology Center (MPC) Cancer Early Detection and Treatment Center (CEDTC) Chongqing University Three Gorges Hospital Chongqing University Chongqing 404100 China; ^3^ Translational Medicine Research Center (TMRC) School of Medicine Chongqing University Chongqing 404100 China; ^4^ National Clinical Research Center for Geriatric Disorders Xiangya Hospital Central South University Changsha Hunan 410008 China; ^5^ Department of Oral and Maxillofacial Surgery Center of Stomatology Xiangya Hospital Central South University Changsha Hunan 410008 China; ^6^ Research Center of Oral and Maxillofacail Tumor Xiangya Hospital Central South University Changsha Hunan 410008 China; ^7^ Insititute of Oral Cancer and Precancerous Lesions Central South University Changsha Hunan 410008 China; ^8^ Department of Dermatology Third Xiangya Hospital Central South University Changsha 410008 China

**Keywords:** lymph node metastatic microenvironment, macrophage, oral squamous cell carcinoma, single cell RNA sequencing, SPP1

## Abstract

Lymph node metastasis, the initial step in distant metastasis, represents a primary contributor to mortality in patients diagnosed with oral squamous cell carcinoma (OSCC). However, the underlying mechanisms of lymph node metastasis in OSCC remain incompletely understood. Here, the transcriptomes of 56 383 single cells derived from paired tissues of six OSCC patients are analyzed. This study founds that CXCR4^+^ epithelial cells, identified as highly malignant disseminated tumor cells (DTCs), exhibited a propensity for lymph node metastasis. Importantly, a distinct subset of tumor‐associated macrophages (TAMs) characterized by exclusive expression of phosphoprotein 1 (SPP1) is discovered. These TAMs may remodel the metastatic lymph node microenvironment by potentially activating fibroblasts and promoting T cell exhaustion through SPP1‐CD44 and CD155‐CD226 ligand‐receptor interactions, thereby facilitating colonization and proliferation of disseminated tumor cells. The research advanced the mechanistic understanding of metastatic tumor microenvironment (TME) and provided a foundation for the development of personalized treatments for OSCC patients with metastasis.

## Graphical abstract

SPP1^+^ TAMs are significantly enriched in metastatic lymph nodes of oral squamous cell carcinoma (OSCC), and it regulates the clonal colonization of CXCR4^+^ malignant epithelial cells by reshaping the metastatic microenvironment.

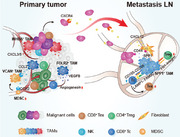

## 1 Introduction

Metastasis is considered the primary contributor to cancer‐related deaths, making it the leading cause of mortality in cancer cases. Lymph node metastasis is crucial for determining the clinical stage and prognosticating outcomes in various malignancies, especially in oral squamous cell carcinoma (OSCC). At the initial presentation, 30–50% of OSCC cancer patients exhibit lymph node metastasis. The 5‐year survival rate for OSCC patients with lymph node metastasis is halved compared to patients without metastasis.^[^ ^1^ ^]^ Pathological confirmation after lymph node dissection is the main method for diagnosis of lymph node metastasis. However, this method has limitations, with a false negative rate of up to 20% due to the presence of micro‐metastases.^[^ ^2^ ^]^ Furthermore, the current focus of anti‐tumor drugs primarily revolves around managing and reducing the size of the primary tumor, without targeting the distant metastasis of the tumor. These facts reinforce the urgent need for extensively investigating the mechanisms underlying lymph node metastasis in OSCC and explore effective diagnostic techniques and potential therapeutic strategies.

The process of distant metastasis in solid tumors is intricate and inefficient, involving several sequential stages, including primary tumor invasion, intravasation, tumor cell survival in the circulation, extravasation, and distant organ colonization.^[^ ^3^ ^]^ The success of tumor metastasis is significantly influenced by the microenvironment at the metastatic site. Tumor cells have developed various strategies to evade immune surveillance at metastatic sites.^[^ ^3^ *^,^* ^4^ ^]^ For instance, in breast cancer, they attract immunosuppressive myeloid cells derived from the bone marrow, which contribute to the formation of a pre‐metastatic niche in the lung.^[^ ^5^ *^,^* ^6^ ^]^ These myeloid cells create a favorable environment for disseminated tumor cells (DTCs) by suppressing local anti‐tumor immunity, promoting extravasation, and providing trophic support to DTCs. However, due to the intricate nature of the metastatic site's ecological niche and the heterogeneity among patients, further investigation is needed to elucidate the mechanisms driving OSCC lymph node metastasis.

Single‐cell RNA sequencing (scRNA‐seq) offers an exceptional opportunity to explore the heterogeneity within OSCC and the ecological niche of its metastatic sites. In this study, we conducted transcriptome profiling of 56383 single cells derived from paired tissues of six OSCC patients. This cohort included two patients without lymph node metastasis and four patients with lymph node metastasis. Through differential analysis of epithelial cell expression profiles, we identified five types of epithelial cells. Furthermore, based on the copy number variation (CNV) analysis, we identified CXCR4^+^ epithelial cells, which express angiogenic and immunosuppressive signals, as highly malignant DTCs. Notably, these DTCs exhibit a propensity for lymph node metastasis.

T cells diversity were assessed, and nine subtypes were identified based on their characteristic gene expression patterns. Our findings revealed that CD8 T cells in metastatic lymph nodes exhibited high expression of genes associated with activation and protein secretion, such as *RPL37A* and *CALM1*. Additionally, these cells expressed exhaustion‐related genes *NR4A2*, suggesting a transition from an activated state to an exhausted state in CD8 T cells within metastatic lymph nodes. Furthermore, SPP1^+^ TAMs were substantially enriched within metastatic lymph nodes. This TAMs remodeled the microenvironment of metastatic lymph node by activating fibroblasts and inducing T cell exhaustion through SPP1‐CD44 and CD155‐CD226, which were conducive to the successful colonization and proliferation of DTCs. Our results provided useful information for the understanding of lymph node metastasis mechanisms and may inform the development of personalized treatment approach to target these for overcoming metastasis to improve clinical outcomes.

## 2 Results

### 2.1 Landscapes of OSCC Metastatic Microenvironment

**Figure 1:**
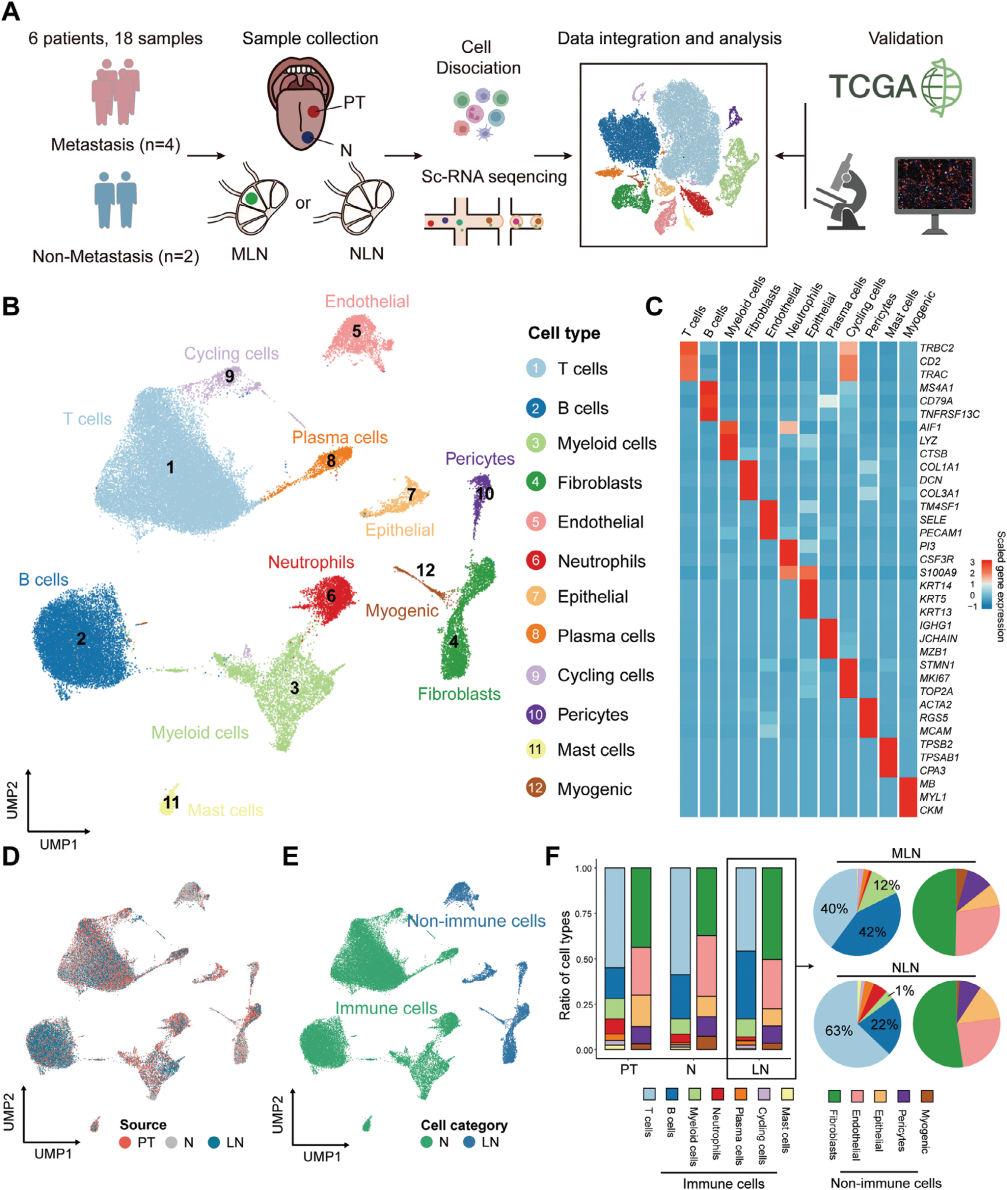
The single‐cell landscape of OSCC patients with lymph node metastases. A) The experimental workflow. B) Uniform manifold approximation and projection (UMAP) plot displays the major cell types, color‐coded by different cell types. C) The heatmap displays the expression of lineage marker genes (rows), with columns representing cell subpopulations. D) UMAP plot color‐coded by sample sources. E) UMAP plot indicates the distribution of immune and non‐immune cells among all cells. Green indicates immune cells, and blue indicates non‐immune cells. F) Histogram indicates the proportion of cells in different sample sources. Non‐immune cells and immune cells are shown in separate histograms.

To gain deeper insights into the microenvironments of OSCC, we performed single‐cell RNA sequencing analysis on samples obtained from six OSCC patients. This analysis included samples from primary tumor tissue (PT), adjacent tumor‐normal tissue (N), metastatic lymph nodes (MLN) from four cases, and non‐metastatic lymph nodes (NLN) from two cases (**Figure** **1**A; Table S1, Supporting Information). After stringent quality filtering and doublet removal (see Experimental Section), we acquired a total of 56 383 cells, comprising 21 266 cells from primary tumor tissue (PT), 15 456 cells from adjacent tumor‐normal tissue (N), and 19661 cells from lymph nodes (LN) (Figure 1B–D).

We utilized the integrated algorithm from the Seurat pipeline for principal component analysis (PCA) and dimensionality reduction. Using lineage‐specific marker genes, we identified six distinct groups of cells, consisting of 25124 T cells (*TRAC*, *TRBC*, and *CD2*), 12719 B cells (*MS4A1*, *CD79A*, and *TNFRSF13C*), 4808 myeloid cells (*AIF1*, *LYZ*, and *CTSB*), 3536 fibroblast cells (*COL1A1*, *COL3A1*, and *DCN*), 2485 endothelial cells (*PECAM1*, *SELE*, and *TM4SF1*), 2430 neutrophils (*PI3*, *S100A9*, and *CSF3R*), 1126 epithelial cells (*KRT5*, *KRT13*, and *KRT14*), and 4155 other clusters (plasma cells, pericytes, myogenic cells, and cycling cells) (Figure 1B,C; Figure S1D and Table S2, Supporting Information). The proportion of immune cells varies greatly in different samples (Figure 1F). Compared to NLN, we observed a significant increase in the proportion of myeloid cells (1% to 12%) and B cells (20% to 42%) in MLN, accompanied by a significant decrease in the proportion of T cells (63% to 40%) (Figure 1F, Figure S1A‐C, Supporting Information).

### 2.2 CXCR4^+^ Malignant Epithelial Cells are the Specific Subcluster of Lymph Node Metastasis

**Figure 2:**
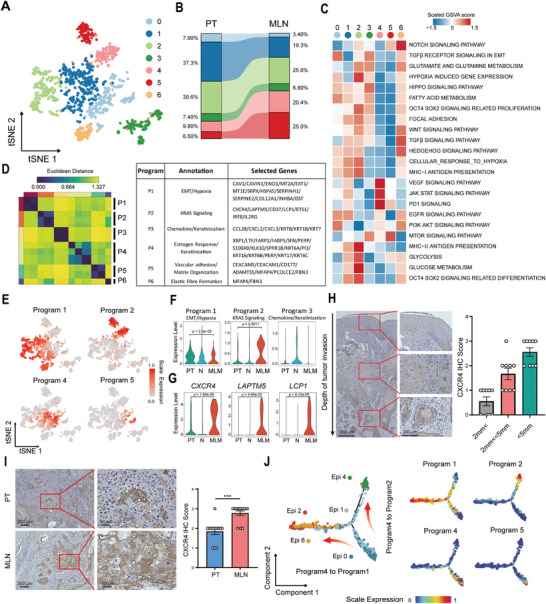
CXCR4^+^ malignant epithelial cells exhibit a propensity to metastasize to lymph nodes. A) tSNE plot of 1126 epithelial cells, color‐coded by subclusters. B) Changes in the proportion of epithelial cell subsets in PT and MLN samples. C) Heatmap shows the selected signaling pathways (rows) that were significantly enriched in GSVA analyses for each epithelial cells cluster (columns). D) Heatmap displays the Euclidean distance of programs across replicates. E) tSNE plot displays the expression levels of different programs. F,G) Violinplots displays the expression of cNMF programs (F) or differential genes (G) in PT, N and MLN samples. The P values were calculated by Wilcoxon test. H) Representative immunohistochemical images of CXCR4 expression with depth of tumor invasion in primary tumors. N = 9. Data presented as mean ± SEM. I) Representative immunohistochemical images of CXCR4 expression in primary tumors (PT) and metastatic lymph nodes (MLN). N = 14. Data presented as mean ± SEM,The P values were calculated by t tests. ^***^ *p* < 0.0001. J) Pseudotime‐ordered analysis of epithelial cells. Epithelial cell subtypes are labeled by colors (left). 2D pseudotime plot displays the gene expression programs identified by NMF analysis in epithelial cells (right).

To investigate the heterogeneity among 1126 epithelial cells, we analyzed their transcriptomic profiles and performed reclustering analysis. Epithelial cells could be categorized into seven clusters based on their expression profiles. (**Figure** **2**A). The proportion of epithelial cells were altered in MLN compared to PT. For instance, the proportion of Cluster 1 has decreased from 37.3% in PT to 19.3% in MLN. Conversely, the proportion of Cluster 4 and Cluster 5 increased in MLN (Cluster 4, MLN 20.4% vs PT 9.8%; Cluster 5, MLN 25.0% vs PT 6.50%) (Figure 2B).

To infer the degree of malignancy for each cluster of epithelial cells, we employed chromosomal copy number variation profile analysis (inferCNV). Compared to the reference data of fibroblast cells, epithelial cells exhibited diverse chromosomal copy number alteration patterns. For instance, frequent detection of amplifications in specific regions of chromosome 6 were observed in clusters 1, 2, 4, 5, and 6, whereas amplifications of chromosome 1 were observed only in cluster 0 and cluster 1 (Figure S2A‐C, Supporting Information). To characterize the potential functions of epithelial cells, we first performed signaling pathway enrichment analyses for each cluster based on the expression levels of genes implicated in each pathway (Table S3, Supporting Information). A distinct pattern of pathway enrichment were observed for each epithelial cluster (Figure 2C). Specifically, pathways associated with epithelial mesenchymal transition (EMT) were significantly enriched in cluster 6, including the “NOTCH SIGNALING PATHWAY”, “WNT SIGNALING PATHWAY”, “TGFβ SIGNALING PATHWAY” and “HEDGEHOG SIGNALING PATHWAY”. These findings indicate that cluster 6 cells are undergoing EMT and possess a high migratory capacity. Cluster 4 exhibited significant enrichment of pathways related to angiogenesis and immunosuppression, including the “VEGF SIGNALING PATHWAY,” “JAK STAT SIGNALING PATHWAY,” and “PD1 SIGNALING”, suggesting a heightened ability to induce angiogenesis and evade immune surveillance. Furthermore, cluster 1 and cluster 2 showed significant enrichment of pathways associated with tumor stemness, including “HYPOXIA INDUCED GENE EXPRESSION,” “GLYCOLYSIS,” “GLUCOSE METABOLISM,” and “OCT4 SOX2 SIGNALING RELATED DIFFERENTIATION”.

Next, to identify heterogeneous expression programs in epithelial cell gene expression profiles, we applied non‐negative factorized matrix algorithm (NMFs) on the total gene expression count matrix of all epithelial cells. After conducting multiple permutation tests and stability assessments, we identified six genes that showed differential expression across various clusters of epithelial cells (Figure 2D). The observed programs were functionally annotated using their top 50 genes, which were ranked based on NMF scores (Figure 2D; Figure S3A,B and Table S4, Supporting Information). Program 1 exhibited enrichment for genes associated with EMT and hypoxia (e.g., CAV1, DST, SERPINH1, and ENO1). These genes were found in cluster 1, cluster 2, and cluster 6. Program 2 was enriched for KRAS and proinflammatory signaling genes (e.g., LCP1, ETS1, and IRF8) and was identified in cluster 4 and cluster 5. Program 3 was enriched for keratins and chemokines associated with normal epithelium (e.g., KRT7, KRT8, KRT18, CCL2, CCL3, and CLL28) and was identified in cluster 3. Program 4 and program 5 were present in cluster 0. Program 4 characterized by estrogen response genes (e.g., XBP1, LTF, FARP1, FABP5, and PERP), while program 5 represented markers of cell adhesion and matrix remodeling processes such as CEACAM5, CEACAM1, and ADAMTS5. The final program 6 encompassed fibrogenesis‐related genes (e.g., MFAP4 and FBN3) (Figure 2D,E). Interestingly, we found that program 1, which is related to EMT and hypoxia, was more expressed in PT than in MLN and N. However, program 2 associated with KRAS signaling was higher expression in MLN than in PT and N (Figure 2F; Figure S4E, Supporting Information).

By comparing the differential genes of epithelial cells in MLN and PT, we found that CXCR4, LCP1, and LAPTM5 are highly expressed in MLN (Figure 2G; Figure S4A,C, Supporting Information). CXCR4 is expressed in a variety of tumor cells and considered to be associated with tumor stemness and metastasis.^[^ ^7^ *^,^* ^8^ ^]^ Up‐regulated genes were significantly enriched in “CELL_SURFACE_INTERACTIONS_AT_THE_VASCULAR_WALL”, “DEGRADATION_OF_THE_EXTRACELLULAR_MATRIX”, “INTEGRIN_CELL_SURFACE_INTERACTIONS” and “MET_PROMOTES_CELL_MOTILITY” pathways, which are related to the ability of tumor cells to penetrate the basement membrane and adhere to peripheral blood vessels to form micro‐metastases in distant places (Figure S4B,D, Supporting Information). Interestingly, we observed CXCR4 expression gradually increased with tumor invasion through the basement membrane (Figure 2H). Moreover, CXCR4 expression was higher in metastatic lymph nodes compared to primary tumors within the same patient (Figure 2I). The results of our analysis suggest that CXCR4^+^ malignant epithelial cells are the specific subcluster of lymph node metastasis.

### 2.3 Evolutionary Trajectories of Malignant Epithelial Cell in OSCC

Tumor cells often undergo changes in gene expression profiles to adapt to the growth environment at metastatic sites. To analyze the evolutionary relationship between tumor cells at the primary site and those that metastasized into the lymph nodes, we performed pseudotime trajectory analysis using Monocle2 (excluding Epi 3 and Epi 5, which were considered normal epithelial cells based on inferCNV results) along trajectories according to their expression and transition profiles. Two distinct developmental trajectories were observed. Epi 0 was located at the initial state of the trajectory, while Epi 4 and Epi 2 were positioned at the terminal states of their respective trajectories. Epi 1 and Epi 6 were located in between, indicating their intermediate functional states (Figure 2J). To better understand the trajectories, we examined the expression of programs identified by NMFs along the trajectories. Interestingly, program P4, characterized by estrogen‐responsive genes, was expressed at the beginning of the trajectories, while program 1 and 2 were respectively expressed at the end of the trajectories (Figure 2F). Additionally, we observed a high association between component 2 and program 2, while component 1 exhibited a negative association with program 1 (Figure S5, Supporting Information). Thus, the states of malignant epithelial cells in OSCC appeared to be shaped by two distinct processes. One process involved estrogen receptor‐driven evolution of tumor cells to an aggressive phenotype regulated by EMT. The other process involved KRAS signaling. These findings are consistent with previous reports that highlight the functional interaction between EMT‐associated transcription factors and estrogen receptor *α* (ER*α*), which leads to the transformation of epithelial‐like cells into mesenchymal cells and promotes distant metastasis.^[^ ^9^ *^–^* ^11^ ^]^ In summary, our results revealed epithelial cell subtypes exhibit different gene expression programs, and are associated with preference for lymph node metastasis of tumor cells.

### 2.4 T Cell Sub‐Clustering and their Functional Changes in MLN and NLN

**Figure 3:**
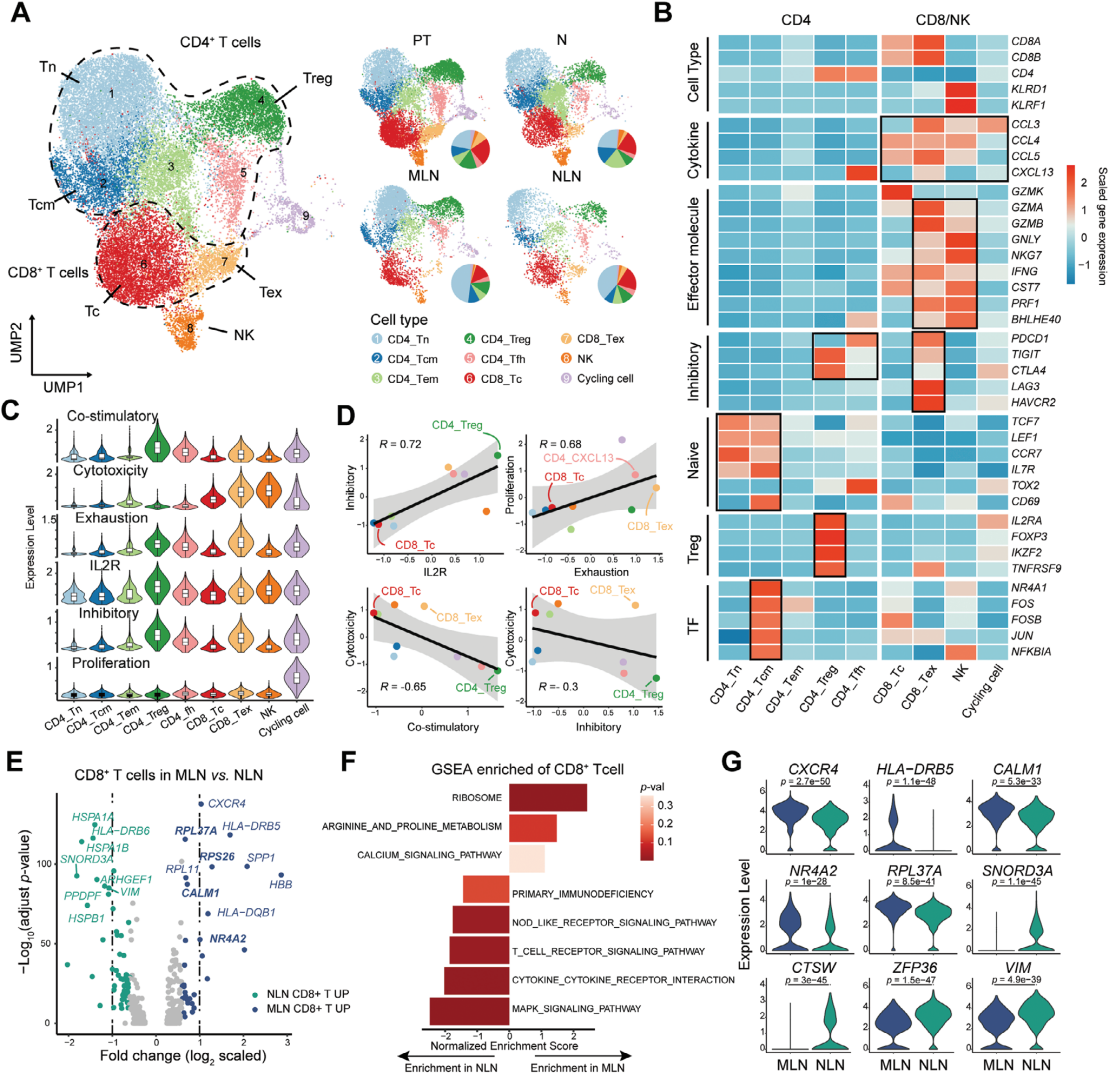
Changes in the transcriptional profile of T cell subsets in the metastatic microenvironment. A) UMAP plot of 25710 T cells displays the components and relative abundance of cells subtypes, color‐coded by subclusters (left) and annotated sample source (right). B) Heatmap displays the scaled mean expression of genes associated with T cell markers, functions and transcription factors (rows) in T cell subclusters. C) Violin plots displays the co‐stimulatory, cytotoxicity, exhaustion, IL2R, inhibitory and proliferation scores of T cell subclusters. Box plots inside the violins indicates the quartiles of corresponding score levels. Endpoints depict minimum and maximum values; central lines denote median values; whiskers denote 1.5 × the interquartile range. D) Significant correlation of scaled scores (inhibitory versus IL2R, proliferation vs exhaustion, cytotoxicity versus co‐stimulatory, and cytotoxicity versus inhibitory). E) Volcano plot displays differentially expressed genes between MLN (blued dots) and NLN CD8^+^ T cells (green dots). The names of the most significant genes are indicated in the plots. F) Two‐sided bar graph displays the enriched activated and inhibited pathways in CD8^+^ T cells in MLN, by GSEA. G) Violin plot indicates the expression levels of differentially expressed genes in CD8^+^ T cells from MLN (blue) and NLN (green) samples. The P values were calculated by Wilcoxon test.

T cells play a significant role in the microenvironment of lymph node metastasis. To explore the composition of T cell subsets in the metastatic microenvironment of OSCC, we analyzed the gene expression of all 25710 T cells and further sub‐clustered them into nine subgroups based on the expression level of classical genes. These subgroups included CD4 naïve (CD4_Tn; *CCR7*, *IL7R*, *TCF7*), CD4 central memory (CD4_Tcm; *JUN*, *FOSB*, *NR4A1*), CD4 effector memory (CD4_Tem; *MALAT1*, *FOS*), regulatory T cell (CD4_Treg; *FOXP3*, *IL2RA*, *TIGIT*), follicular helper T cell (CD4_Tfh; *CXCL13*, *MAF*, *CD200*), cytotoxic T cell (*CD8_Tc; GZMK*, *TNFSF9*, *NKG7*), exhausted T cell (CD8_Tex; *GZMB*, *LAG3*, *HAVCR2*), NK cell (*KLRD1*, *KLRF1*, *NKG7*), and cycling cell (*TOP2A*, *MKI67*) (**Figure** **3**A; Figure S6A, Supporting Information). The majority (48.03% in MLN and 38.64% in NLN) of CD4_Tn were found in LN, whereas the majority of CD4_Treg (15.66%), CD8_Tc (23.39%) and CD8_Tex (8.63%) were located in PT (Figure 3A; Figure S6B, Supporting Information). It is noteworthy that CD8_Tex exhibited high expression of effector molecules, including *GZMA*, *GZMB*, and *IFNγ*. Additionally, they expressed a range of immune checkpoint molecules, including *PDCD1*, *LAG3*, and *HAVCR2* (Figure 3B; Figure S6A, Supporting Information), indicating that they represented an intermediate cell in the transition from activated effector T cells to a state of exhaustion.

To evaluate the functional status of T cells, we calculated co‐stimulatory, cytotoxicity, exhaustion, IL2R, inhibitory and proliferation scores for all T cell clusters (Table S8, Supporting Information). Notably, CD4_Treg demonstrated higher scores in both co‐stimulatory and IL2R, which is associated with their competitive binding to IL2 and inhibition of effector T cell activation. Consistent with our previous findings, both CD8_Tex and NK cells exhibited higher cytotoxicity scores, while CD8_Tex cells also showed higher exhausted scores (Figure 3C). Combined with scores for other functional modules of T cells, we observed a positive correlation between Inhibitory and IL2R scores (R = 0.72), as well as between Proliferation and Exhausted scores (R = 0.68). Additionally, we observed a negative correlation between Cytotoxicity and Co‐stimulatory scores (R = −0.65), as well as between Cytotoxicity and Inhibitory scores (R = −0.3) (Figure 3D).

Differentially expressed gene (DEG) analysis revealed that the top upregulated genes in CD8^+^ T cells in MLN included *RPL37A*, *RPL11*, *RPS26*, *and CALM1* (Figure 3E,G; Table S10, Supporting Information). The upregulated genes were enriched in calcium and ribosome pathways, which are related to T cell activation and protein secretion.

This suggests that CD8^+^ T cells in MLN may be activated, leading to the upregulation of ribosomal genes involved in the synthesis of effector proteins (Figure 3F). However, NR4A2, a transcription factor known to cooperate with NFAT to decrease the activity of AP‐1 and promote the exhaustion state of CD8^+^ T cells,^[^ ^12^ *^,^* ^13^ ^]^ was found to be upregulated in MLN (Figure 3E,G). Based on these findings, we speculate that CD8^+^ T cells in MLN may exist in a transitional state between activation and exhaustion, which may contribute to the survival of metastatic cells.

CD4^+^ T cells play a crucial role in maintaining immunological tolerance and homeostasis. In our study, we observed upregulation of *TSC22D3* and *TXNIP* in MLN (Figure S7A,C and Table S11, Supporting Information). TSC22D3, known for its role in immune regulation, inhibits the expansion and activation of T cells by suppressing both T‐cell receptor‐induced interleukin‐2/interleukin‐2 receptor expression and NF‐kappaB activity.^[^ ^14^ ^]^ TXNIP can bind to TRX1, a crucial component involved in maintaining cellular redox homeostasis, thereby inhibiting T cell activity and proliferation.^[^ ^15^ ^]^ Gene set enrichment analysis (GSEA) revealed significant enrichment of TGF_BETA_SIGNALING_PATHWAY, NOD_LIKE_RECEPTOR_SIGNALING_PATHWAY and MAPK_SIGNALING_PATHWAY in MLN (Figure S7B, Supporting Information). The data indicate a potential decrease in CD4^+^ T cell proliferation and proinflammatory cytokine secretion within the metastatic lymph nodes (MLNs), which may consequently impair the effector functions of CD8^+^ T cells.

### 2.5 Changes in B Cell Subsets in Metastatic Lymph Nodes

A total of 13913 B cells were identified and grouped into eight clusters based to the expression of characteristic genes (Figure S8A,B, Supporting Information). C1 highly expressed B cell activation related genes CD69, CD83, and MARCK and were identified as activated B cells. C2 was identified as naïve B cells because it expressed FCER2 and IL4R.^[^ ^16^ ^]^ C3 uniquely expresses the transcription factor XBP1 and multiple immunoglobulin genes (IGHG1, IGHA1, IGLC3, and IGHGP, etc.) and is annotated as a plasma cell. C5 expressed CXCR5 and IRAG2 and was identified as a memory B cell. In particular, we identified two populations of transitional cells (C4: pre‐pro B cells and C5: pre‐activated B cells) according to their high expression of CD7, IL7R TNFAIP3 (for pre‐pro B cells) and BHLHE41, ZMAT3 (for pre‐activated B cells).^[^ ^17^ ^]^ Interestingly, we observed that the proportion of activated B cells in lymph nodes with tumor metastasis are lower compared to lymph nodes without metastasis. However, the proportion of naive B cells exhibited an opposite trend (Figure S8A, Supporting Information). This suggests that B cell‐mediated humoral immunity may be impaired in lymph nodes with tumor metastasis.

### 2.6 The Accumulation of SPP1^+^ TAM in OSCC Lymph Node with Metastasis

**Figure 4:**
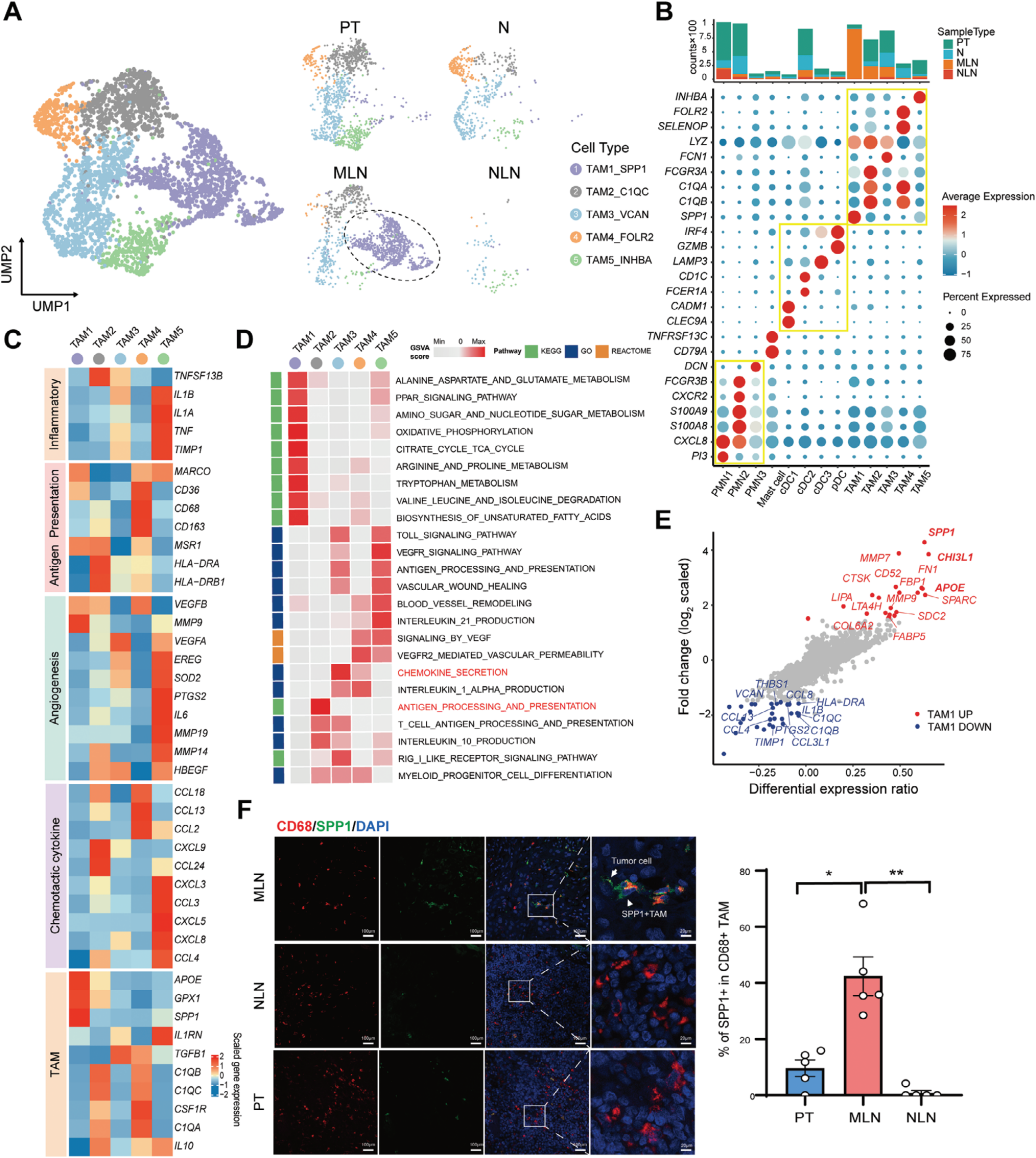
High levels of lipid metabolism SPP1^+^ TAM are enriched in metastatic lymph nodes. A) UMAP plot of 3243 TAMs displays the components and relative abundance of cells subtypes, color‐coded by subclusters (left) and annotated sample source (right). B) Differential expression of functional state signatures for myeloid cells subclusters and stacked bar plot displays the cell number of myeloid cells with distinct states, color‐coded by their sample source. Dot size: percent of cells expressing gene and dot color: scaled average expression. C) Heatmap displays the scaled mean expression of genes associated with functional gene of TAMs D) Heatmap displays the selected signaling pathways (rows) that were significantly enriched in GSVA analyses for each TAMs cluster (columns). Green, blue, and orange purple squares on the left column represent the results derived from KEGG, GO, and REACTOME signaling pathways analysis, respectively. E) Volcano plot displays differentially expressed genes between TAM1_SPP1 (red dots) and the remaining TAMs (blue dots). F) Representative IF staining images displays the expression intensity of SPP1 and CD68 in MLN, NLN and PT (left). Histogram illustrated the fraction of SPP1^+^ CD68^+^ TAMs, in matched PT, MLN, and NLN pairs. Data presented as mean ± SEM, N = 5,The P values were calculated by one‐way ANOVA test. ^*^ *p* < 0.01, ^**^ *p* < 0.001.

A total of 6891 myeloid cells were identified and categorized into 13 distinct subsets, including one subset for mast cells, five subsets for TAMs, three subsets for polymorphonuclear neutrophils (PMNs), three subsets for conventional dendritic cells (cDCs), and one subset for plasmacytoid dendritic cells (pDCs) (**Figure** **4**B; Figure S9A, Supporting Information). Macrophages accounted for over half of the myeloid cells. Notably, the composition of myeloid‐derived cells significantly differed across the microenvironments of N, PT, and MLN (Figure S9B, Supporting Information). For example, the proportion of neutrophils decreased from 40.77% in PT to 32.88% in the N, and was found to be less than 1% in MLN. In contrast, the fraction of TAMs increased from 36.22% in PT to 81.66% in MLN, while accounting for only 18.94% in NLN (Figure S9B, Supporting Information). These findings suggest a critical involvement of myeloid‐derived cells in the development of OSCC and lymph node metastasis. Among the increased TAMs, five distinct subtypes were identified, each exhibiting unique transcriptomic profiles (Figure 4A). TAM1 showed significant enrichment in MLN and exhibited high expression of M2 macrophage genes, including *SPP1*, *APOE*, and *GPX1*, as well as angiogenesis‐related genes such as *VEGFB* and *MMP9* (Figure 4A–C; Figure S9C, Supporting Information). Both TAM2 and TAM4 expressed *C1QA*, *C1QB*, *C1QC*, and *CSFR1*, indicating their monocyte‐derived macrophage origin. TAM2 displayed elevated expression of inflammation‐associated chemokines (*TNFSF13*, *CXCL9*, *CCL24*) and major histocompatibility complex (MHC) molecules (Figure 4B,C). TAM4 exhibited high expression of FOLR2 and genes associated with antigen processing and presentation, including *CD36*, *CD68*, and *CD163*. TAM3 was characterized by its expression of *FCN1* and *VCAN*. Lastly, TAM5, enriched in the primary tumor (PT), exhibited high expression of various immunosuppressive factors, including *IL1A*, *IL1B*, *IL1RN*, and *IL6* (Figure 4B,C).

To characterize the potential functions of TAMs, we initially conducted gene set variation analysis (GSVA) for each TAMs cluster based on the expression levels of genes. We observed a distinct pattern of pathway enrichment for each TAMs cluster (Figure 4D; Table S6, Supporting Information). Particularly, the metabolic level of TAM1_SPP1 was significantly changed, “ALANINE_ASPARTATE_AND_GLUTAMATE_METABOLISM”, “GLYCOLYSIS_GLUCONEOGENESIS” and “BIOSYNTHESIS_OF_UNSATURATED_FATTY_ACIDS” were highly enriched in TAM1_SPP1. Glutamine contributes to the replenishment of the tricarboxylic acid (TCA) cycle, thereby promoting the glycosylation of lectin/mannose receptors, which are among the most characteristic markers of M2 macrophage polarization.^[^ ^18^ ^]^ Glucose oxidation has been demonstrated to be required for the early differentiation of M2 macrophages.^[^ ^19^ ^]^ What's more, enhanced fat metabolism is also thought to promote the transition of pro‐inflammatory M1 macrophages to anti‐inflammatory M2 macrophages.^[^ ^20^ ^]^ TAM1_SPP1 is considered to have a high metabolic level of the M2 subpopulation. Antigen recognition processing and presentation signaling pathways were enriched in TAM3_VCAN and TAM5_INHBA, including “TOLL_SIGNALING_PATHWAY” and “ANTIGEN_PROCESSING_AND_PRESENTATION”. Interestingly, “VEGFR_SIGNALING_PATHWAY” and “BLOOD_VESSEL_REMODELING” were also enriched in TAM5_INHBA suggested that TAM5_INHBA has strong angiogenesis ability. “T_CELL_ANTIGEN_PROCESSING_AND_PRESENTATION” and “INTERLEUKIN_10_PRODUCTION” are simultaneously enriched in TAM2_C1QC indicated it can inhibit T cell effector functions. To explore polarization state of TAMs, we calculated the scores related to macrophage angiogenesis, inflammatory cytokine secretion, and M1 or M2 polarization state based on their expression level using the AddModuleScore function implemented in Seurat software. We observed the TAM3_VCAN and TAM5_INHBA have higher M1 scores. Conversely, TAM1_SPP1, TAM2_C1QC and TAM4_FOLR2 had lower M1 scores and higher M2 scores (Figure S9E, Supporting Information). Consistent with previous results, TAM5_INHBA had the highest angiogenic level and chemokine score (Figure S8E and Table S9, Supporting Information). Our results suggest multiple polarization states of TAMs in OSCC primary and metastatic sites, which is associated with primary tumor progression and lymph node metastasis.

TAM1_SPP1 is considered to play an important role in OSCC lymph node metastasis due to uniquely existed in LNM microenvironment. We found that TAM1_SPP1 not only expressed a variety of metabolism‐related genes (*LPL*, *FBP1*, *FABP5*, *APOE*, *APOC1*), but also highly expressed *CHI3L1*, *CD52*, *GPNMB* and multiple matrix protease genes (*CTSK*, *MMP7*, *MMP9*) (Figure 4E; Figure S8C and Table S7, Supporting Information). CHI3L1, CD52, and GPNMB as secreted proteins can be autocrine or paracrine by macrophages. CHI3L1 can facilitate tumor invasion and metastasis by upregulating the expression levels of matrix metalloproteinase (MMP) genes in various tumor cells^[^ ^21^ *^,^* ^22^ ^]^ and CD52 can inhibit Toll‐like receptor activation of NF‐κB to suppress inflammation.^[^ ^23^ ^]^ What more, GPNMB can exert effects on tumor cells that promote their survival, enhance the expansion of cancer stem cells, and facilitate the acquisition of a metastatic phenotype.^[^ ^24^ ^]^ Using the TCGA‐HNSC cohort, we found that high expression of TAM1_SPP1 related genes was associated with poorer prognosis (P = 0.0033) (Figure S9F, Supporting Information). Immunofluorescence (IF) imaging revealed that SPP1 was predominantly expressed in CD68^+^ macrophages in the MLN of OSCC patients, whereas it was nearly absent in CA and NLN (Figure 4F). Our results suggest that TAM1_SPP1, a subpopulation of TAMs characterized by high levels of glycolysis and associated elevated levels of lipid metabolism and glutamate metabolism, plays an important role in promoting lymph node metastasis of tumor cells.

### 2.7 SPP1^+^ TAMs are Derived from VCAN^+^ Inflammatory Monocytes

**Figure 5:**
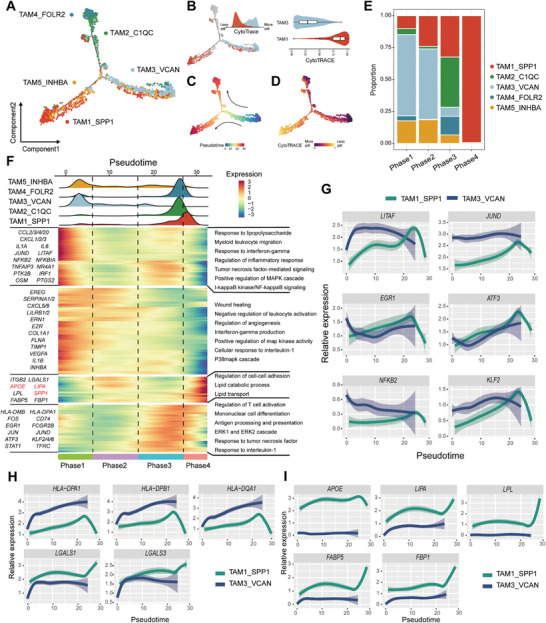
SPP1^+^ TAMs are derived from VCAN^+^ inflammatory monocytes. A) Pseudotime‐ordered analysis of five TAMs by Monocle. B) Pseudotime‐ordered analysis marked by TAM1_SPP1 and TAM3_VCAN (Left). CytoTRACE score in TAM3 versus TAM1 (Right). C) 2D pseudotime plot displays the pseudotime score identified by Monocle. D) 2D pseudotime plot displays the CytoTRACE score identified by CytoTRACE. E) Histogram displays the cell distribution of TAMs. TAMs subtypes labeled by colors. F) Heatmap displays the dynamic changes in gene expression along the pseudotime (lower panel). The distribution of TAMs subtypes during the transition (divided into four phases), along with the pseudo‐time. Subtypes were labeled by colors (upper panel). 2D plots showed the loess regression‐smoothened dynamic expression (y‐axis) of transcription factor G), histocompatibility complexes or immunosuppressive molecules H), and lipid metabolism genes I) during the TAMs transitions along the pseudotime. Green, TAM1_SPP1; blue, TAM3_VCAN.

TAMs can originate both from tissue‐resident macrophages (TRMs) or newly recruited monocytes that subsequently differentiate into macrophages. We next explored the polarization state and cell transitions in OSCC‐infiltrated TAMs by inferring the cellular trajectories using Monocle2 and CytoTRACE (**Figure** **5**A–D; Figure S10, Supporting Information). We projected the CytoTRACE score onto the trajectory derived by Monocle2 (Figure 5B–D). Interestingly, TAM3_VCAN exhibits a lower CytoTRACE score and is located at one end of the trajectory, while TAM1_SPP1 is positioned at the other end of the trajectory with a higher CytoTRACE score (Figure 5D). Based on the analysis results of Monocle2 and CytoTRACE, we determined TAM3_VCAN as the starting point of the trajectory (Figure 5C). The transition through an intermediate state characterized by TAM5_INHBA or TAM2_C1QC cells, and finally reach TAM1_SPP1 or TAM4_FOLR2 (Figure 5A–D; Figure S10, Supporting Information).

We next investigated the transcriptional changes associated with transitional states. We observed that TAMs clusters could be categorized into four phases (Figure 5E). TAM3_VCAN were predominantly phase 1, characterized by upregulated expression of *IL1A*, *IL6*, *NFKB2*, *JUND*, and *IRF1* (Figure 5F). Pathway analysis revealed that signaling pathways associated with myeloid cell migration, interferon responses, and inflammation, including the MAPK and NF‐κB pathways, were significantly enriched in phase 1. This suggests that the cells likely originated from circulating inflammatory monocytes. Additionally, the cGAS‐STING pathway, which can be activated by diverse immunogens within the tumor microenvironment, appears to be broadly engaged. (Figure 5F). Phase 2 mainly consists of TAM3_VCAN, TAM5_INHBA and TAM1_SPP1 and characterized by upregulation of *CXCL5*, *CXCL8*, *EREG*, *ERN1* and *VEGFA*. CXCL5 and CXCL8 can combine with CXCR2 on tumor cells and vascular endothelial cells to promote tumor metastasis and angiogenesis.^[^ ^25^ *^‐^* ^27^ ^]^ TAMs‐derived EREG acts as a ligand for EGFR and is associated with tumor invasion.^[^ ^28^ ^]^ What's more, ERN1 is thought to be associated with the polarization of TAMs toward the M2 phenotype.^[^ ^29^ ^]^ These genes were enriched in wound healing, angiogenesis, and negative regulation leukocyte activation pathways. In this stage, there is a decrease in the proportion of TAM3_VCAN and a corresponding increase in TAM1_SPP1 compared to phase 1, which may indicate a transition from the inflammatory mononuclear phenotype of TAM3_VCAN to the proangiogenic and immunosuppressive functions of TAM1_SPP1. Cells in phase 3 demonstrated the highest expression levels of HLA class II related genes include *HLA‐DMB*, *HLA‐DPA1* and *CD74*. Pathways including “Regulation of T cell activation” and “Antigen processing and presentation” were enriched in this phase. It's worth noting that cells in phase 3 also expressed ATF3, a key transcriptional regulator that inhibits the inflammatory response and promotes the transition of macrophages from the M1 phenotype to the M2 phenotype.^[^ ^30^ *^,^* ^31^ ^]^ This finding indicated that phase 3 cells may be in an intermediate state of macrophage polarization, which not only have the function of antigen processing and presentation to activate adaptive immune cells, but also have the anti‐inflammatory function of maintaining immune homeostasis. Consistently, cells in phase 3 are composed of multiple types of TAMs, also suggested that they are in the intermediate state of the macrophage polarization trajectory. Phase 4, as the terminal state of the trajectory, is mainly composed of TAM1_SPP1 and characterized by enriched in lipid transport and metabolism‐related pathways.

Due to TAM3_VCAN and TAM1_SPP1 represent the two ends of the polarization state of macrophages in OSCC, we nest analyze the transcription factors (TFs) expressed in these cells. Our analysis revealed *ATF3* and *KLF2* are significant upregulated in phase 1 to phase 3, however, downregulated in phase 4 (Figure 5G). Like ATF3, KLF2 is thought to negatively regulate the inflammatory response of macrophages by inhibiting the transcriptional activity of NF‐kB.^[^ ^32^ ^]^ Consistently, *NFKB2* was downregulated in this process (Figure 5G). *LITAF*, an important mediator of the LPS‐induced inflammatory response distinguished from NF‐kB pathway,^[^ ^33^ ^]^ was downregulated in TAM3_VCAN while upregulated in TAM1_SPP1 (Figure 5G). This finding indicated that TAM1_SPP1 has a different inflammatory response program regulated by LITAF than TAM3_VCA. What's more, TAM1_SPP1 has higher lipid metabolism gene expression (*LIBA*, *LPL* and *FBP1*), upregulated up to three times compared with TAM3_VCAN (Figure 5I). Interestingly, HLA class II related genes (*HLA‐DPA1*, *HLA‐DPB1* and *HLA‐DQA1*) were significantly upregulated in TAM3_VCAN while downregulated in TAM1_SPP1 (Figure 5H). However, immunosuppressive molecules *LGALS1* and *LGALS3* were upregulated in TAM1_SPP1 suggested that it could reshape the immune microenvironment by inhibiting the activation of T cells^[^ ^34^ *^,^* ^35^ ^]^ (Figure 5H).

We conclude that two distinct activation trajectories of TAMs in OSCC primary and metastatic sites. Compared with its precursor inflammatory monocyte TAM3_VCAN, TAM1_SPP1 enriched in metastatic lymph nodes has lower antigen‐presenting ability, but potentially remodels the immune microenvironment by suppressing T cell activation, possibly favoring early tumor cell lymph node colonization.

### 2.8 SPP1^+^ TAMs Remodel the Lymph Node Microenvironment by CD155‐CD226 and SPP1‐CD44 Ligand Receptor Pair

**Figure 6:**
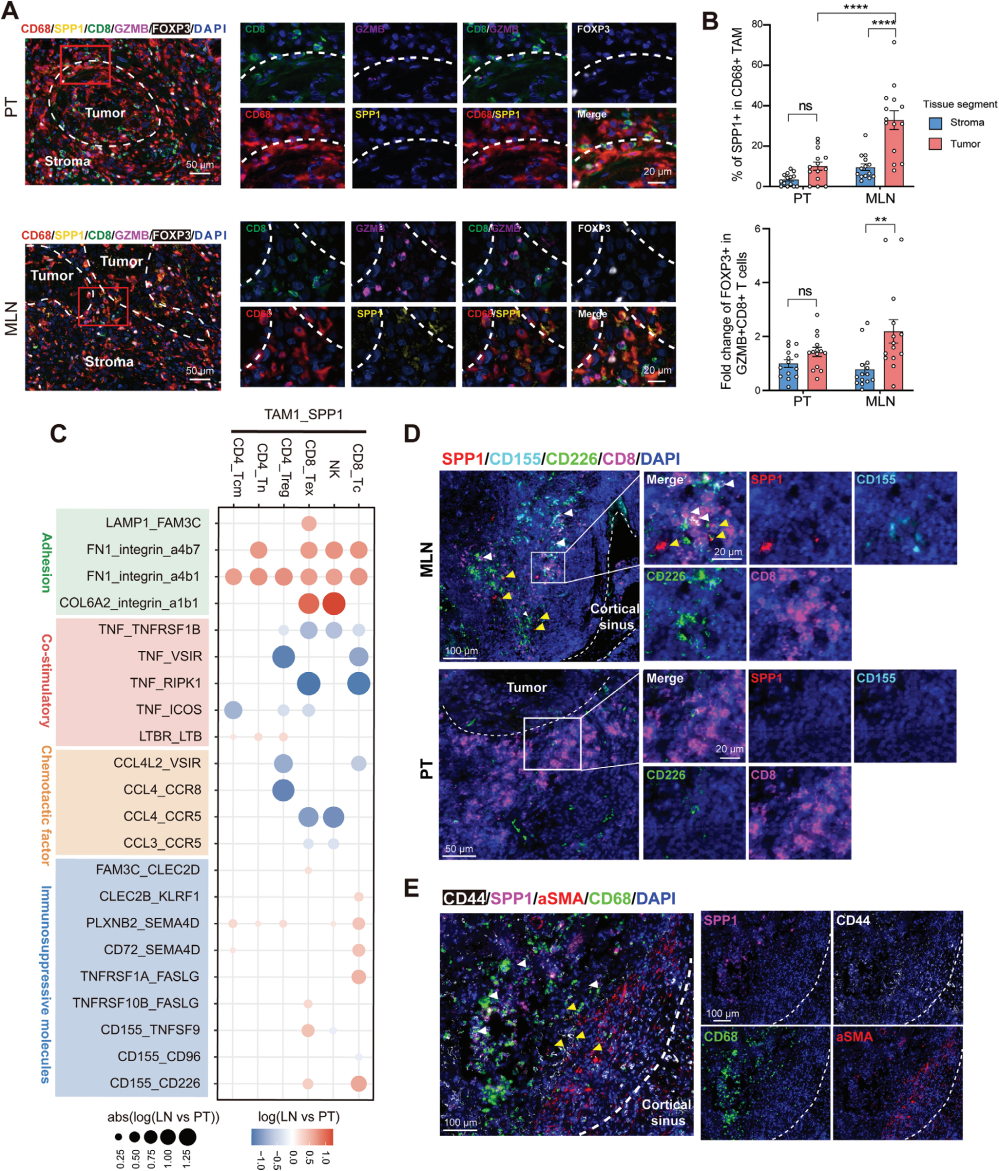
Different tumor immune phenotypes between primary tumor and metastatic lymph node shaped by SPP1^+^ TAM. A) Representative images of mIHC staining for the Treg cells (FOXP3^+^), Cytotoxic T cells (CD8^+^ GZMB^+^), and SPP1^+^ CD68^+^ TAMs in PT and MLN. Images are representative of ten biological replicates. B) Top, the fraction of SPP1^+^ CD68^+^ TAMs in both tumor nest, and stroma between PT and MLN (N = 14). Bottom, the fold change of FOXP3^+^ Treg to GZMB^+^ CD8^+^ T cells ratio in both tumor nest, and stroma between PT and MLN (N = 14). Data presented as mean ± SEM. The P values were calculated by one‐way ANOVA test. ns, not significant, ^**^ *p* < 0.001, ^****^ *p* < 0.00001. C) Dot plots displays selected ligand‐receptor interactions between TAM1_SPP1 and T cells, as well as NK cells. The ligand‐receptor interactions and cell‐cell interactions are indicated at columns and rows, respectively. The log10(LN versus PT) were indicated by colour heatmap in one‐sided permutation test. The absolute value of log10(LN versus PT) were indicated by circle size. Different color boxes at the left represent different function modules of receptor‐ligand interactions. D) Representative images of multiplex IHC staining for the SPP1^+^ CD155^+^ TAMs and CD8^+^ CD226^+^ T cells in PT and MLN. Dotted lines indicated the boundary of cortical sinus and tumor, respectively. White arrows indicates CD226^+^ CD8^+^ T cells; Yellow arrows indicates SPP1^+^ CD155^+^ TAMs. E) The spatial position of SPP1^+^ CD68^+^ macrophages and CD44^+^ aSMA^+^ fibroblasts. Dotted lines indicates the boundary of cortical sinus. White arrows indicates SPP1^+^ CD68^+^ macrophages; Yellow arrows indicates CD44^+^ aSMA^+^ macrophages.

To evaluate the impact of enriched SPP1^+^ TAMs in metastatic lymph nodes on immune surveillance, we performed multiplex immunohistochemistry (IHC). The following combinations of antibodies for multiplexed IF assays were used: CD8 and GZMB (for effector CD8^+^ T cells), CD68 and SPP1 (for SPP1^+^ macrophages), and FOXP3 (for Treg T cells) (**Figure** **6**A; Figure S12, Supporting Information). These multiplexed IF staining were performed on ten matched primary tumor and metastatic lymph node from OSCC patients, one to three regions per sample. Using these data, we observed that SPP1^+^ TAM is more enrich in tumor area in MLN than PT (Figure 6B). Consistently, the ratio of FOXP3^+^ cell to GZMB^+^ CD8^+^ T cell in MLN tumor area was higher than PT, suggesting that the immunosurveillance of the metastatic area in the lymph nodes was suppressed. (Figure 6B).

To investigate the mechanisms underlying TAM1_SPP1‐induced immunosuppression in metastatic lymph nodes, we analyzed ligand‐receptor interactions between TAM1_SPP1 and lymphocyte subsets from OSCC PT and LN using CellPhoneDB. We observed intensive cellular interactions among the TAM1_SPP1, CD8_Tc, NK, CD8_Tex, CD4_Treg, CD4_Tn and CD4_Tcm, mediated by immunosuppressive, co‐stimulatory, chemokine, and adhesion molecules (Figure 6C). Compared to the PT ecosystem, the interaction between TAM1_SPP1 and lymphocytes in the LN ecosystem demonstrated a significant increase in the intensity of adhesion and immunosuppressive molecule expression. However, the intensity of co‐stimulatory molecules and chemokines reduced in LN ecosystem. TAMs remodel the extracellular matrix by secreting FN1 (fibronectin‐1), binds to integrin receptors on the lymphocyte membrane, and limits its contact with tumor cells to avert T cell‐mediated tumor elimination.^[^ ^36^ *^,^* ^37^ ^]^ CD155, as an immunosuppressive molecule, can bind to the co‐stimulatory receptor CD226 and TNFSF9 on the surface of CD8^+^ T cell membrane to disable it^[^ ^38^ *^,^* ^39^ ^]^ (Figure 6C). We also found that co‐stimulatory interaction and chemotaxis between TAM1_SPP1 and T cells in LN were attenuated, including TNF_ICOS, CD72_SEMA4D, CCL4_CCR5 and CCL3_CCR5 (Figure 6C). Using mIHC technology, we observed co‐localization of SPP1^+^ CD155^+^ macrophages with CD226^+^ CD8^+^ T cells, which were near the cortical sinuses of lymph nodes, the channels through which lymphatic return flows (Figure 6D). However, this colocalization is missing in PT (Figure 6D).

By examining the gene expression profiles of various cell types in our dataset, we found that fibroblasts highly express CXCL12, which can bind CXCR4 in the malignant epithelial cells (Figure S11A, Supporting Information). Interestingly, our analysis demonstrated increased interaction between fibroblasts and malignant epithelial cells in lymph nodes mediated through the CXCL12‐CXCR4 ligand‐receptor axis (Figure S11E, Supporting Information). To explore the effect of SPP1^+^ TAM enriched in lymph nodes on fibroblasts, we analyzed the cellular communication between TAMs and fibroblasts. Notably, cellular interactions between SPP1^+^ TAMs and fibroblasts via SPP1‐CD44 were increased in lymph nodes (Figure S11D, Supporting Information). CD44 is a key molecule for fibroblast activation, which enhances CXCL12 expression through ERK phosphorylation.^[^ ^40^ *^,^* ^41^ ^]^ Using mIHC technology, we observed colocalization of CD44^+^ fibroblasts with SPP1^+^ macrophages (Figure 6E). We also observed colocalization of CXCL12^+^ fibroblasts with CXCR4^+^ CK17^+^ single tumor cells in the subcapsular sinus, the initial site of disseminated tumor cell arrival (Figure S13, Supporting Information).

In summary, our analysis tentatively suggests that SPP1^+^ TAMs might play a role in remodeling the microenvironment through various ligand‐receptor interactions, such as CD155‐CD226 and SPP1‐CD44, potentially contributing to the chemotactic colonization and survival of CXCR4^+^ malignant epithelial cells.

### 2.9 Heterogeneity of Macrophages Across Various Metastatic Sites in Different Types of Cancer

**Figure 7:**
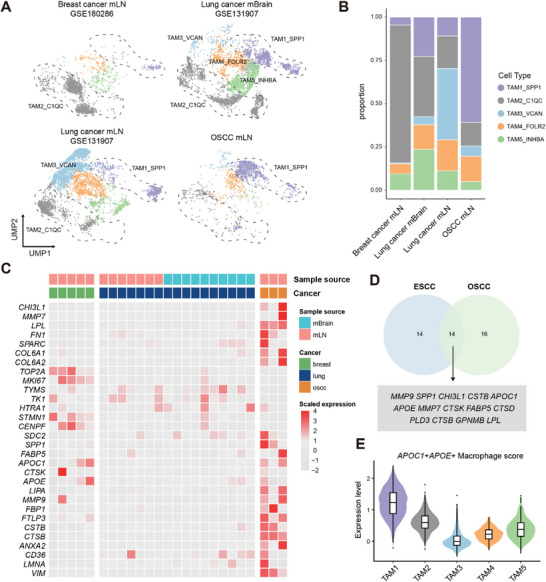
Heterogeneity of macrophages across various metastatic sites in different types of cancer. A) UMAP plot illustrates the distribution of macrophages in various tumor metastases, color‐coded by subclusters. B) The bar plot displays the proportion of macrophage subpopulations across different tumor metastasis sites. C) The selected genes expression in TAM1_SPP1 from different sample sources. Each column in the heatmap represents a patient. Labels above the heatmap indicates the sample source and tumor type. D) Venn diagram illustrates the intersection of highly expressed genes in *APOC1^+^ APOE^+^* macrophages in ESCC and TAM1_SPP1 in OSCC. The specific 14 intersection genes are listed below. E) The violin plot displays the expression scores of *APOC1^+^ APOE^+^* macrophages in TAM subpopulations in our manuscript. Box plots inside the violins indicate the quartiles of corresponding score levels.

To investigate macrophage enrichment in the microenvironment of metastatic sites across other cancers, we integrated metastasis data from lung and breast cancers.^[^ ^42^ *^,^* ^43^ ^]^ Joint alignment of these datasets identified the presence of subpopulations of TAMs across metastatic microenvironment of different cancers (**Figure** **7**A). We found that TAM1_SPP1 is present in lymph node metastasis and brain metastasis of lung cancer, as well as lymph node metastasis of breast cancer. TAM1_SPP1 in various tumor metastasis microenvironments shows consistent gene expression profiles (Figure 7C), including genes like *LPL, FN1, SPARC, SDC2, FABP5, APOE, CD36*, etc. However, TAM1_SPP1 is not a prominent subpopulation in the metastatic microenvironment of lung cancer and breast cancer (Figure 7A,B). Conversely, TAM2_C1QC is enriched in breast cancer metastatic lymph nodes, while TAM3_VCAN is enriched in lung cancer lymph nodes (Figure 7A,B), suggesting functional diversity among macrophages across metastatic niches of varying cancer types. Furthermore, we compared the highly expressed genes of *APOC1^+^ APOE^+^* macrophages enriched in esophageal squamous cell carcinoma (ESCC) lymph nodes,^[^ ^44^ ^]^ which exhibit a similar tendency to lymph node metastasis as OSCC. Interestingly, up to 14 genes are expressed simultaneously in *APOC1^+^ APOE^+^* macrophages and TAM1_SPP1 (Figure 7D,E), such as *SPP1, CHI3L1, and APOC1*.

## 3 Discussion

A thorough comprehension of the tumor immune microenvironment is essential for enhancing the effectiveness of immunotherapy in OSCC. While multiple studies have focused on the TME in primary OSCC, the TME in OSCC lymph node metastases remains enigmatic.^[^ ^45^ *^‐^* ^47^ ^]^ We observed diverse transcriptional patterns in tumor cells within primary tumors and metastatic lymph nodes. Tumor cells with hypoxia‐induced EMT phenotype were enriched in PT and MLN, which is consistent with previous study reporting that EMT transition of tumor cells can be induced by the hypoxic environment of the primary tumor, resulting in distant metastasis.^[^ ^48^ ^]^ We also found that tumor cells with high KRAS signal expression increased from PT to MLN, and their high expression of *CXCR4* was associated with directional chemotaxis and metastasis of tumor cells to lymph nodes.^[^ ^49^ ^]^ Tumor heterogeneity is an important reason for the failure of targeted drug therapy. Our study revealed a subpopulation of CXCR4^+^ malignant epithelial cells with high metastatic potential and provided new insights into targeting this subpopulation to prevent lymph node metastasis.

Exhaustion of T cell in tumor‐draining lymph nodes is thought to precede tumor metastasis. Forming a supportive niche helps metastatic tumor cells escape immune surveillance. Previous studies have shown that myeloid derived suppressor cells (MDSC) can induce the formation of an immunosuppressive niche in draining lymph.^[^ ^50^ *^‐^* ^52^ ^]^ Our study is the first to identify SPP1^+^ TAMs are enriched in metastatic LNs. It high expression of various immunosuppressive molecules, including *SPP1*, *CHI3L1*, *and SPARC*. We found that SPP1^+^ TAMs developed from VCAN^+^ TAMs by pseudo‐time analysis. Our results also revealed that SPP1^+^ TAMs induce T cell exhaustion through multiple ligand receptor pairs such as CD155‐CD226. CD155, a member of poliovirus receptor–related (PRR) family, has been identified as the ligand of CD226 on T cells. Recent studies have shown that tumor cell‐derived CD155 can bind to CD226 on the surface of CD8^+^ T cells to induce its ubiquitination and degradation, thereby inducing T cell exhaustion.^[^ ^39^ ^]^ Our study also prompted that SPP1^+^ TAMs activated fibroblasts through ligand receptors such as SPP1‐CD44. This increased expression of CXCL12 in fibroblasts and chemotactic colonization of CXCR4^+^ malignant epithelial cells to lymph nodes.

**Figure 8:**
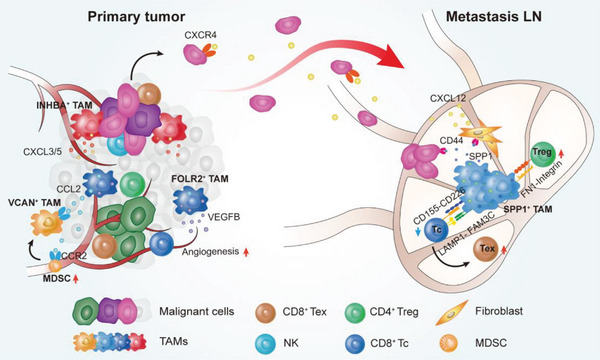
Schematic diagram of cross‐talks among multiple immune and tumor cells in the lymph node metastasis microenvironment of OSCC. FLOR2^+^ TAM, VCAN^+^ TAM and INHBA^+^ TAM are enriched in primary tumor. VEGFB from FLOR2^+^ TAM and CXCL3/5 from INHBA^+^ TAM promote angiogenesis. CCL2 from FLOR2^+^ TAM chemoattracts MDSC and induces its transformation into VCAN^+^ TAM. The malignant epithelial cells of the primary tumor with EMT phenotype highly express CXCR4 and metastasize to the draining lymph nodes. SPP1^+^ TAM are enriched in metastasis lymph nodes. SPP1 from SPP1^+^ TAM promotes the survival and proliferation of tumor cells by binding to CD44. SPP1 also can bind the CD44 in fibroblast to promote the secretion of CXCL12, which can chemoattract CXCR4^+^ tumor cells. CD8^+^ Tc cells interact with SPP1^+^ TAM through LAMP1‐FAM3C and CD155‐CD226, which might induce T cell exhaustion. SPP1^+^ TAM interact with CD4^+^ Treg through FN1‐ Integrin, which increase its adhesion to Treg. The intensive cell‐cell interactions among SPP1^+^ TAM, Treg cells, CD8^+^ Tc cells, and malignant cells foster an immune‐suppressive niche for OSCC lymph node metastasis.

The heterogeneous function of tumor‐associated macrophages in tumor initiation, progression, and metastasis have been extensively studied in primary TME of various cancer types (e.g., colorectal, lung, and liver cancers).^[^ ^53^ *^‐^* ^56^ ^]^ Studies have shown that SPP1^+^ macrophages are thought to play pro‐angiogenic and matrix‐remodeling functions in primary tumors,^[^ ^54^ *^,^* ^55^ ^]^ but remains unclear in lymph node metastatic microenvironment. In this study, we not only furtherly revealed that SPP1^+^ TAMs are major components of the OSCC metastatic microenvironment but also identified SPP1^+^ TAMs as a unique subpopulation that promotes OSCC lymph node metastasis, reshapes the microenvironment through ligands such as CD155‐CD226 and SPP1‐CD44 (**Figure** **8**). Our study serves as a valuable foundation for further investigations. The insights gleaned from these findings could aid in refining our understanding of the tumor microenvironment and in developing diverse therapeutic strategies targeting metastasis‐related immune cell subsets and tumor cells, with the goal of inhibiting tumor growth and overcoming metastasis.

## 4 Experimental Section

### Patient Samples Collect

Six patients with pathologically diagnosed of OSCC were enrolled in this study. Eighteen independent surgical resected specimens were collected from six OSCC patients, including primary tumor (PT), adjacent nontumor tissue (N) and lymph node with metastasis (MLN, four cases) or without metastasis (NLN, two case). All clinical samples were collected at the Xiangya Hospital of Central South University (Hunan Province, China) and their clinical information are summarized in Table S1 (Supporting Information). Informed consent was obtained from all patients before the collection of samples. The samples used in this study were approved by the Committees for Ethical Review of Research at Central South University (202211261).

### Single Cell Suspension Preparation, Library Construction, and Sequencing

To obtain a single cell suspension, the fresh tissue samples surgical resected from OSCC patients were washed in ice‐cold RPMI1640 and dissociated using Multi Tumor Dissociation Kit, human (cat. 30‐095‐929) from Miltenyi Biotec according to manufacturer's instructions. Single‐cell RNA‐Seq libraries were prepared using BD Rhapsody system (BD Genomics, Menlo Park, CA) according to Single cell 3′ whole transcriptome amplification (WTA) with sample multiplexing kit (SMK) protocol. Library construction and sequencing were performed as previously described.^[^ ^57^ ^]^

### Single‐Cell rna‐seq Data Processing

The BD Rhapsody Analysis pipeline was used to process fastq data. The output was converted to a Seurat object using the R Seurat package (version 4.3.0) for downstream analysis.^[^ ^58^ ^]^ Use DoubletFinder to remove double cells for each sample.^[^ ^59^ ^]^

Low quality cell removal criteria: nUMI > = 300, nGene > = 200 and mitoRatio > = 0.20. For the remaining cells, use the Seurat standard process for dimensionality reduction and de‐batching. Cell clusters were identified using the FindClusters function in Seurat. Resolutions from 0.1 to 0.5 were used, and the most appropriate resolution was selected based on the results. Differential genes in each population of cells were identified using Seurat's FindAllMarkers function with default parameters. The cluster with multiple well‐defined marker genes of different cell types were considered cell contamination and eliminated before downstream analysis.

### Copy Number Variants (CNV) Analysis

To explore the heterogeneity of copy number alterations existing in epithelial cells of primary tumor and lymph node metastasis site of OSCC, inferCNV (version 1.16.0) was applied to estimate the changes of large‐scale chromosomal copy number variants in a single epithelial cell.^[^ ^60^ ^]^ Single cell data from fibroblasts were used as a control reference.

### Gene Expression Programs (GEPs) Analysis of Epithelial Cell Heterogeneity

To delve deeper into the heterogeneous functions of epithelial cells, consensus non‐negative matrix factorization (cNMF) was employed to dissect distinct GEPs related to cell function, status and fate.^[^ ^61^ ^]^ The top 50 genes were selected based on their contribution scores to specific GEPs as the representative gene set for conducting pathway enrichment analysis (Table S5, Supporting Information).

### Evolutionary Trajectory Analysis by Monocle2

Monocle2 (version 2.22.0)^[^ ^62^ ^]^ was used to infer the evolutionary trajectories and functional changes of TAMs and epithelial cells. The data from the indicated clusters identified by Seurat were input directly into Monocle2. Next, the differentialGeneTest function was used in Monocle2 to order cells based on DEGs between clusters. The top significant genes (q value < 0.01) were used for ordering in all instances. The cell differentiation trajectory was inferred using Monocle2's default parameters after dimension reduction and cell ordering.

### Calculation of Cellular Functional Module Scores

The functional module scores were calculated for each subcluster using the AddModuleScore function in Seurat. The involved gene features of each module were downloaded from previous study^[^ ^63^ ^]^ and their average expression of single cells for each subcluster were calculated.

### Cellular Communication Analysis

CellPhoneDB^[^ ^64^ ^]^ and CellChat^[^ ^65^ ^]^ was used to investigate the cell‐cell communications in OSCC microenvironment.

For CellPhoneDB, the gene expression matrixes of T, myeloid, and malignant epithelial cells were selected as input for the CellPhoneDB analysis. This study only considered ligand pairs expressed in more than 20% of the subpopulation. The default parameters of CellPhoneDB was used and selected the interaction pairs with biologically relevance (P‐value > 0.1).

CellChat was utilized with recommended parameters to quantify cellular communication probabilities. Ligand receptor pairs were analyzed differentially expressed in primary tumor (PT) and lymph nodes (LN).

### Pathway Enrichment Analysis

To compare signaling pathway enrichment between the two subclusters, the fgsea package (version 1.20.0) was used with default parameters. This involved testing the enrichment of Gene Ontology and KEGG terms using a pre‐ranked gene list based on fold changes. To explore the heterogeneity of epithelial cell or TAMs, gene set variation analysis (GSVA, version 1.42.0) was performed, using 18 hallmark pathways or KEGG and GO terms.

### Immunofluorescence (IF) Staining

IF staining was performed on formalin‐fixed, paraffin‐embedded sections. After dewaxed and rehydrated, the slides were incubated with the primary antibodies to SPP1 (Cat.AF1433, R&D Systems); CD68 (Cat.ab213363, Abcam) at 4 °C overnight. After washing thoroughly, Alexa Fluor 594‐ or 488‐ conjugated secondary antibodies (Invitrogen) were used to incubate the slides. Images were captured with a ZEISS LSM900 fluorescence confocal microscope.

### Multiplexed Immunofluorescence (mIHC) Staining

For mIHC staining, 5 µm thick FFPE sections of OSCC tissues were stained with the Opal 7 colour fluorescent IHC Kit (PerkinElmer, Massachusetts, USA) according to protocols. The primary antibodies were: CD8 (Cat. 85336S, CST), SPP1 (Cat.AF1433, R&D Systems), CD68 (Cat. ab213363), FOXP3 (Cat. ab20034, Abcam) and GZMB (Cat. ab255598, Abcam). DAPI was used for nuclear counterstaining. The slides were finally mounted with antifade reagent (G1401, Servicebio, Wuhan, China). PhenoImager HT was used to capture the images and identify all markers of interest. The 1 to 3 representative fields of the whole‐slide scan images were selected and quantitatively analyzed by ImageJ. The staining and analysis results of the mlHC were also checked by two certified pathologists.

### Statistics Analysis

This study conducted a range of statistical tests using R (version 4.1.3), including Wilcoxon rank tests, log‐rank tests, Pearson correlation tests, and Kruskal–Wallis tests. Log‐rank tests were conducted for overall survival analysis. Linear regression analysis was applied for correlation test. Multigroup analysis used one‐way ANOVA test of variance.

## Conflict of Interest

The authors declare no conflict of interest.

## Author Contributions

L.D. and S.H. contributed equally to this work. L.D. was responsible for the overall experimental design, and performed all data analysis and processing. L.D. wrote and revised the paper. L.D., L.J., and S.H. supervised the sample collection and performed image acquisition. X.L., S.P., and L.Z. participated in the discussion during the experiment. X.C., A.M., and M.Y. supervised the study. All of the authors have read and approved the paper.

## Supporting information

Supporting Information

Supporting Information

## Data Availability

The data that support the findings of this study are openly available in [Genome Sequence Archive (GSA)] at [https://ngdc.cncb.ac.cn/omix/], reference number [6117].
