# Supplementary material for: SPP1+ TAM Regulates the Metastatic Colonization of CXCR4+ Metastasis‐Associated Tumor Cells by Remodeling the Lymph Node Microenvironment
Source: Adv Sci (Weinh). 2024 Sep 5;11(44):2400524. doi: 10.1002/advs.202400524 (PMC11600252; doi:10.1002/advs.202400524)
Supplement: Supplementary file 1 — Supporting Information [file ADVS-11-2400524-s002.pdf]

ADVANCED SCIENCE
Open Access

Supporting Information

for *Adv. Sci.*, DOI 10.1002/advs.202400524

SPP1$^+$ TAM Regulates the Metastatic Colonization of CXCR4$^+$ Metastasis-Associated Tumor Cells by Remodeling the Lymph Node Microenvironment

Liang Dong, Shujun Hu, Xin Li, Shiyao Pei, Liping Jin, Lining Zhang, Xiang Chen, Anjie Min* and Mingzhu Yin**

Title:

SPP1+ TAM regulates the metastatic colonization of CXCR4+ metastasis-associated tumor cells by remodeling the lymph node microenvironment.

Supplemental figures and legends

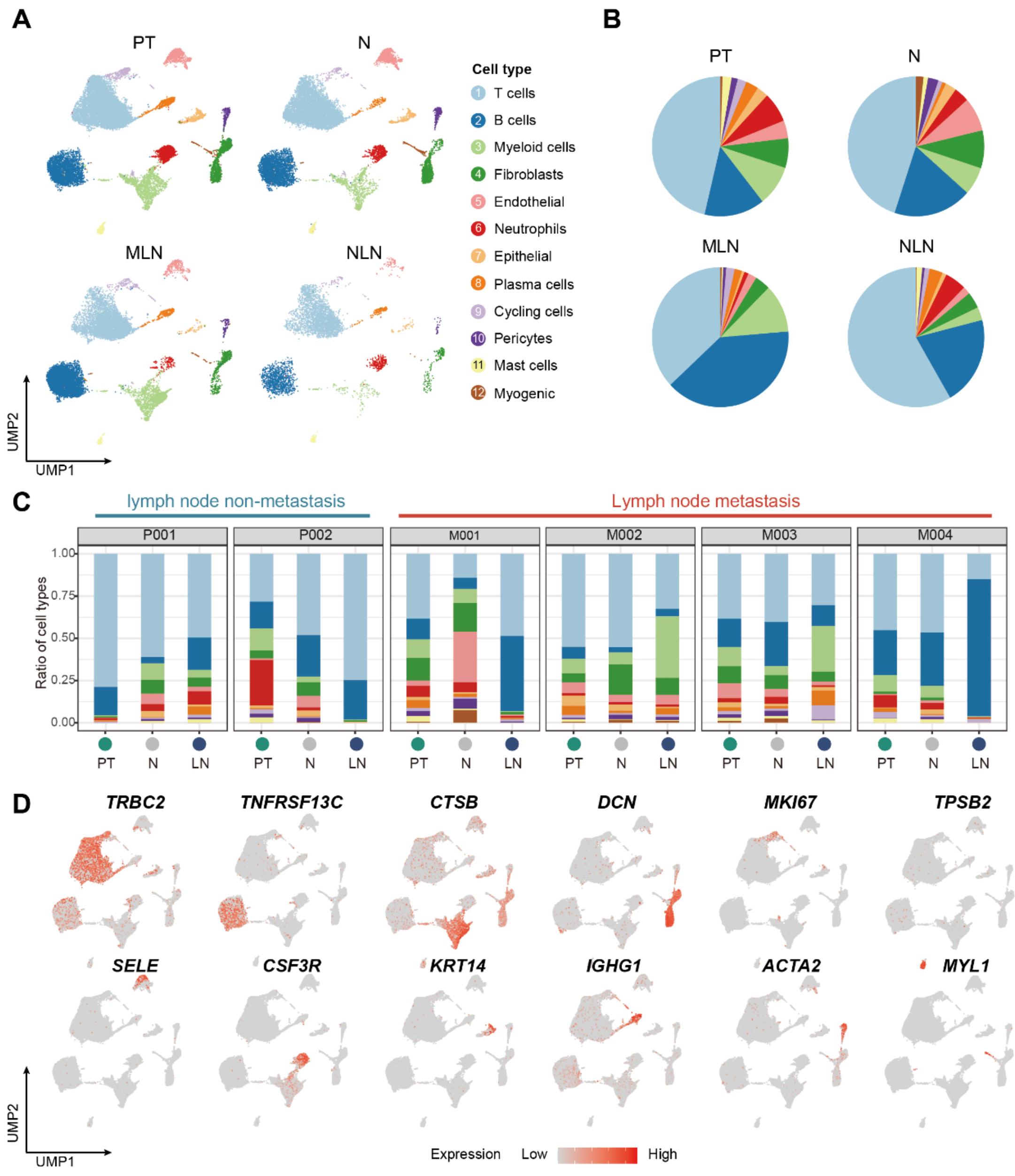

Figure S1. The distribution and characteristic of cell subsets in

the immune microenvironment of OSCC. (A) UMAP plot divided by sample source and color-coded based on the major cell lineage. (B) Proportions of subsets in different sample sources. (C) The bar chart displays the proportion of different cell subpopulations in samples from various patients, color-coded based on the major cell lineages as shown in (A). (D) UMAP plot displays the expression levels of subpopulation marker genes.

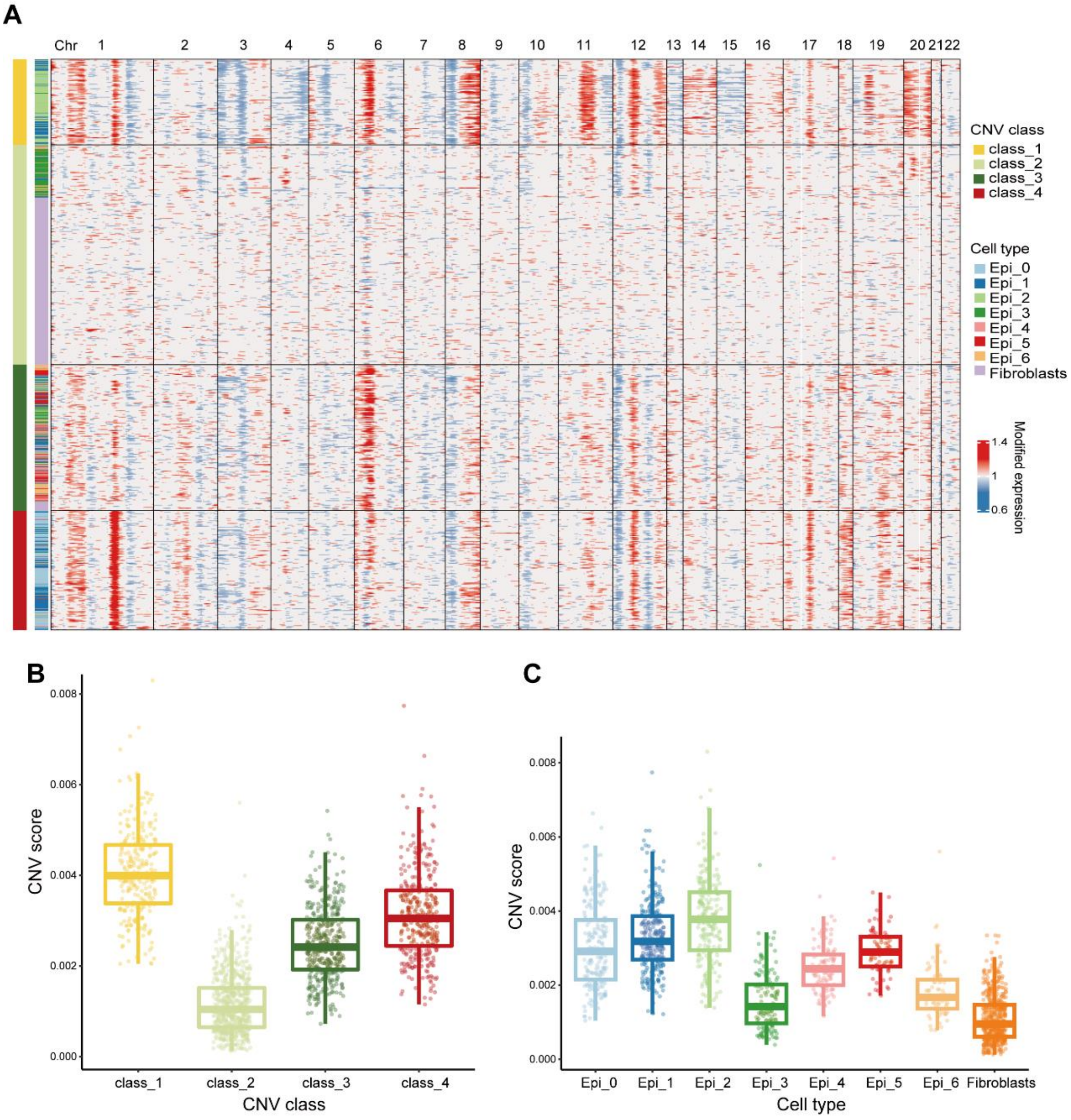

Figure S2. The CNV analysis of epithelial cells. (A) Heatmap

displays the large-scale CNVs for epithelial cells (rows along y-axis). Red: amplification and blue: deletion. Epithelial cells from different subclusters and the range of different chromosomes were labeled by different color bars on the left and top to the heatmap, respectively. (B) Boxplots displays scores of four populations of cells classified according to CNV scores. (C) Boxplots displays CNV scores of epithelial and fibroblasts. A point represents a single cell.

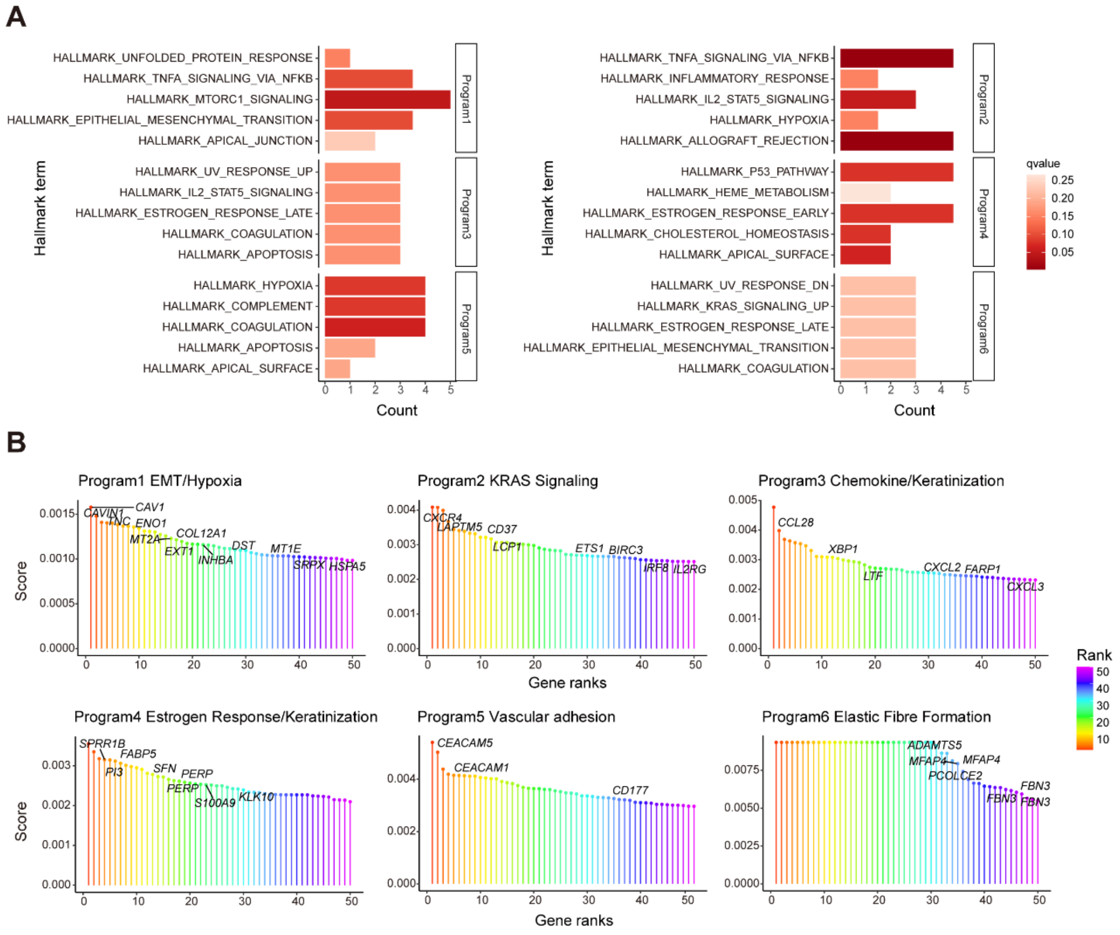

Figure S3. Annotation and characterization of 6 gene expression programs in Epithelial cells. (A) Gene enrichment analysis of top50 genes ranked by contribution score in each gene expression programs. (B) The distribution of contributing score for

top50 genes in each gene expression programs.

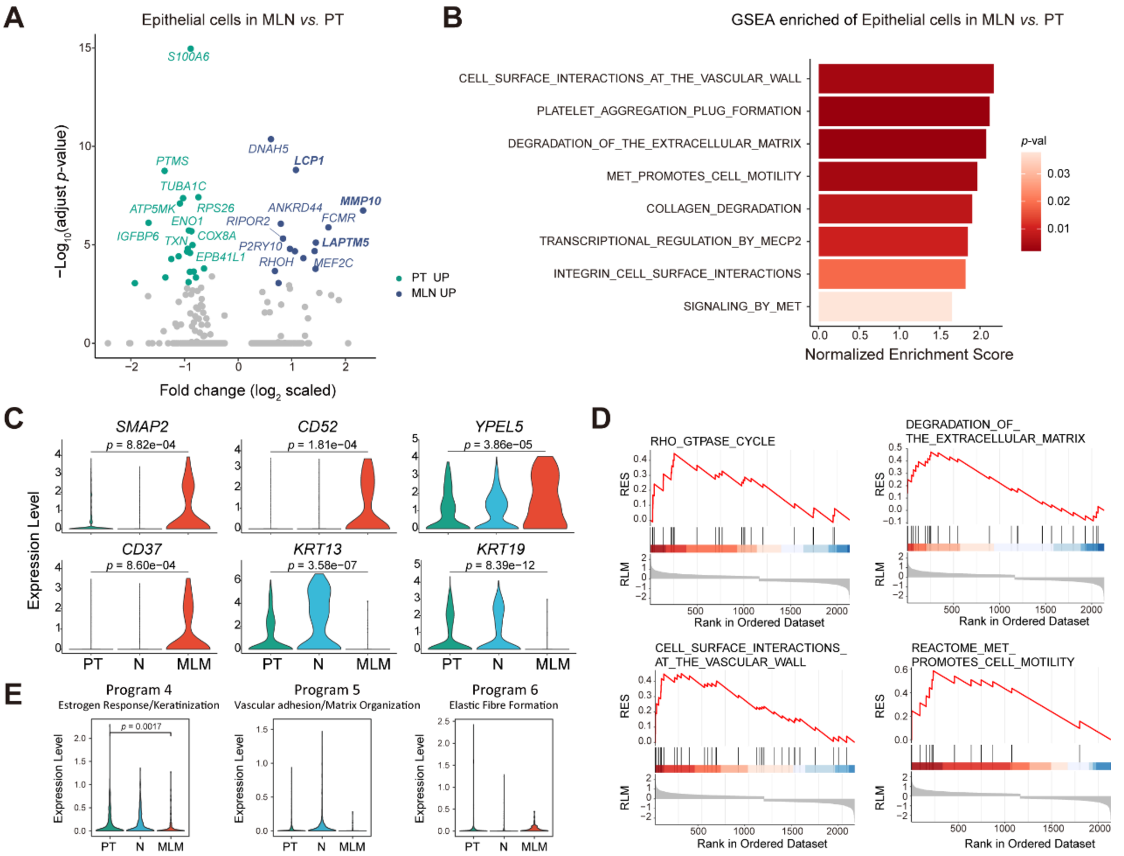

Figure S4. The characteristic of epithelial cells in metastatic lymph nodes and primary tumors. (A) Volcano plot indicates differentially expressed genes between MLN (blued dots) and PT epithelial cells (green dots). (B) Bar plot displays the enriched activated and inhibited pathways in epithelial cells in MLN, by GSEA. (C) Violin plot indicates the expression levels of differentially expressed genes in epithelial cells from different samples. The P values were calculated by Wilcoxon test. (D) Enriched pathway of differentially expressed genes in MLN epithelial cells comparing with PT epithelial cells. (E) Violinplots displays the expression of cNMF programs in PT, N and MLN samples. PT, primary tumors; N, normal

tissue; MLN, metastatic lymph nodes. The P values were calculated by Wilcoxon test.

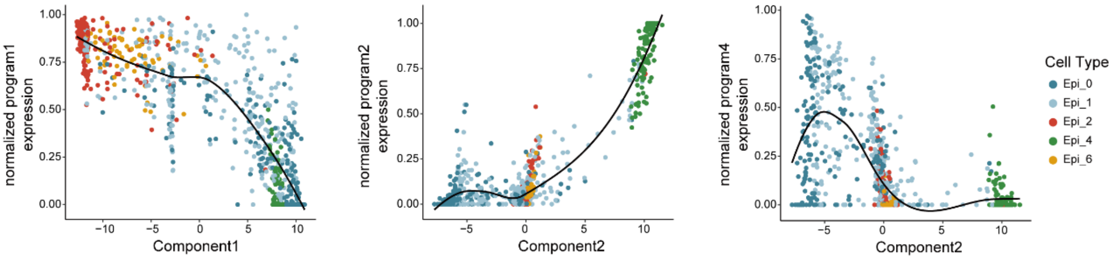

Figure S5. Expression changes of gene expression programs with temporal components identified by Monocle2. A point represents a single cell. Colors represent different cell subsets.

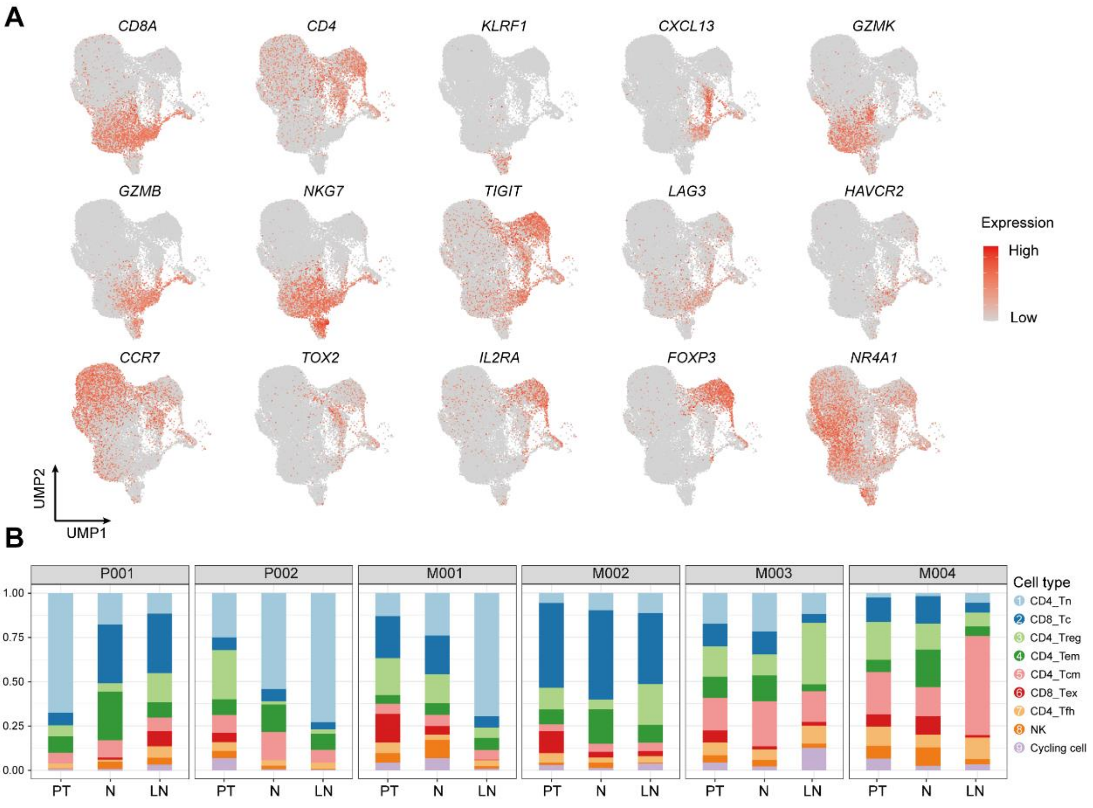

Figure S6. Percentages and characterization of T cell subsets. (A) UMAP plot displays the expression levels of marker genes in T cell subsets. (B) Histogram indicates the proportion of T cells in different sample sources of each patient.

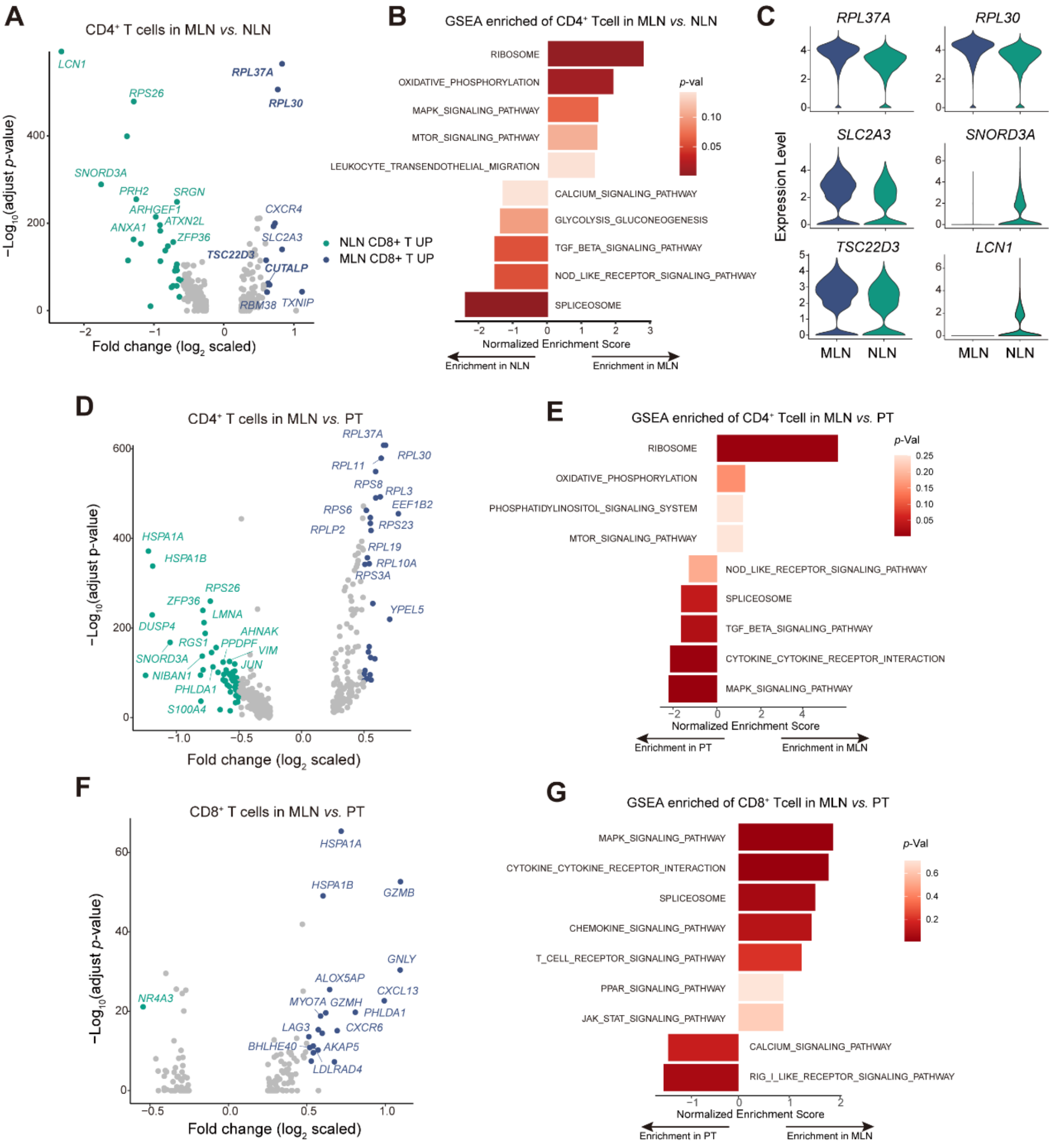

Figure S7. Characterization of T cell subsets. (A) Volcano plot displays differentially expressed genes between MLN (blued dots) and NLN (green dots) in CD4$^+$ T cells. (B) Two-sided bar graph displays the enriched activated and inhibited pathways in CD4$^+$ T cells in MLN, by GSEA. (C) Violin plot indicates the expression levels of differentially expressed genes in CD4$^+$ T cells from MLN (blue) and NLN (green) samples. Volcano plot displays differentially

expressed genes between MLN (blued dots) and PT (green dots) in CD4⁺ T cells (D), CD8⁺ T cells (F). Two-sided bar graph displays the enriched activated and inhibited pathways in MLN, CD4⁺ T cells (E) and CD8⁺ T cells (G).

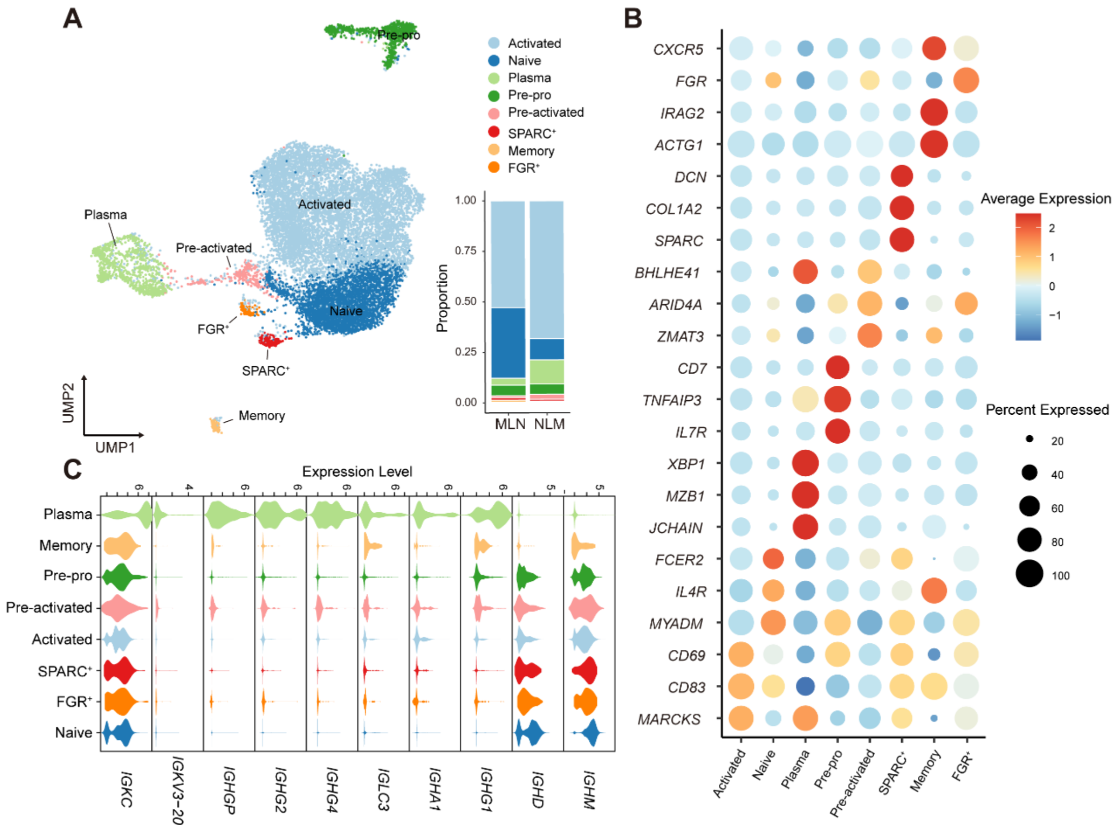

Figure S8. B cell subsets in the OSCC metastatic microenvironment. (A) UMAP plot of 13,913 B cells displays the components and relative abundance of cells subtypes, color-coded by subclusters. The bar plot displays the distribution of B cell subsets in metastatic (MLN) and non-metastatic (NLN) lymph nodes. (B) Dot plot displays characteristic gene expression of B cell subsets. (C) Violin plot displays the expression of different immunoglobulin genes in B cell subsets.

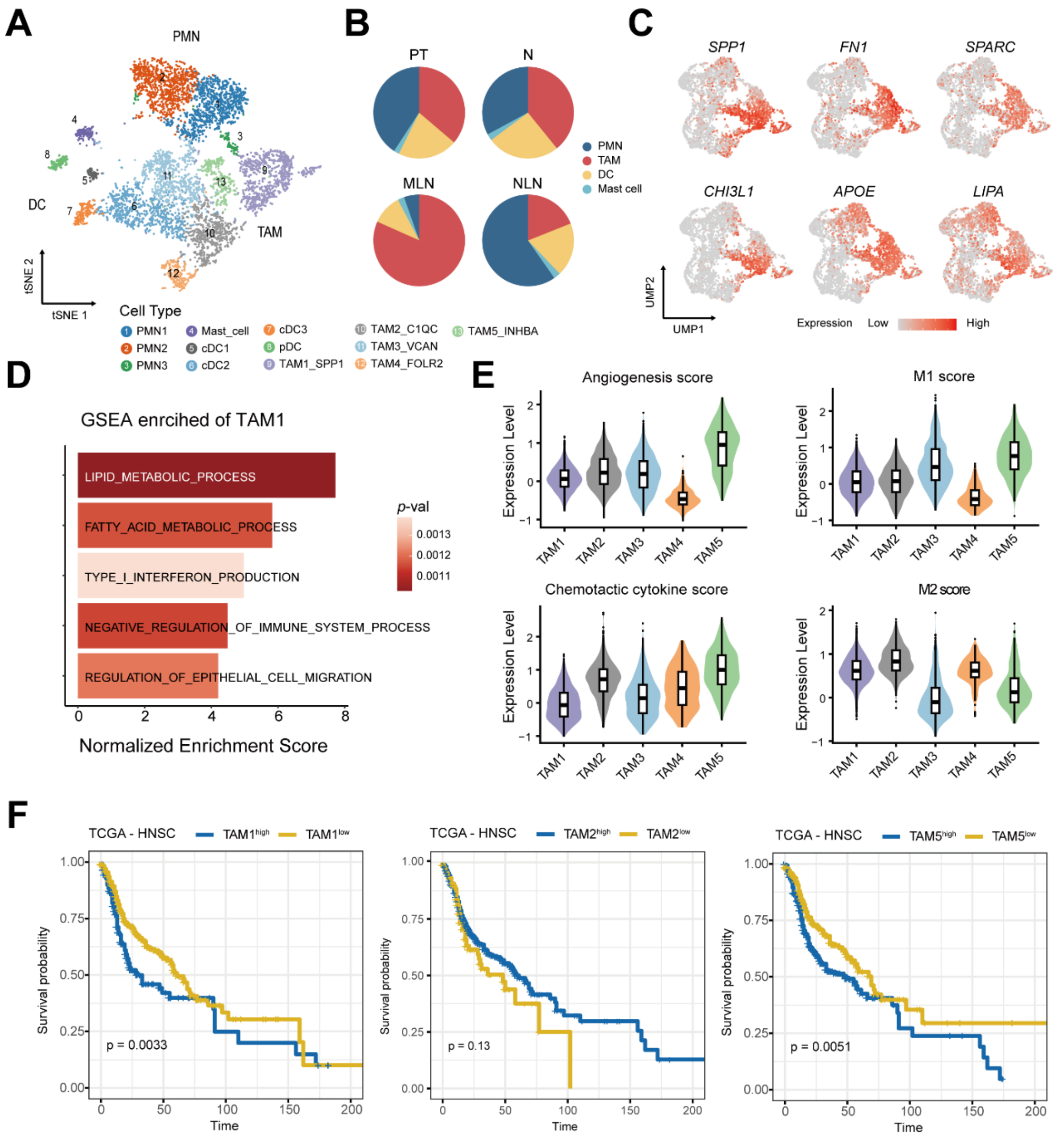

Figure S9. The characteristic of TAMs in OSCC. (A) tSNE plot of 6,891 myeloid cells indicates the components and relative abundance of cells subtypes, color-coded by subclusters. (B) Proportions of myeloid cell subsets in different sample sources. (C) UMAP plot displays the expression levels of marker genes in TAM1_SPP1. (D) Pathway enrichment of genes upregulated by TAM1_SPP1. (E) Violinplot displays functional gene set scores in TAMs subset. Box plots inside the violins indicated the quartiles of

corresponding score levels. (F) Kaplan–Meier curves for overall survival in the 514 patients in TCGA-HNSC cohort stratified according to TOP50 upregulated genes in TAM1_SPP1.

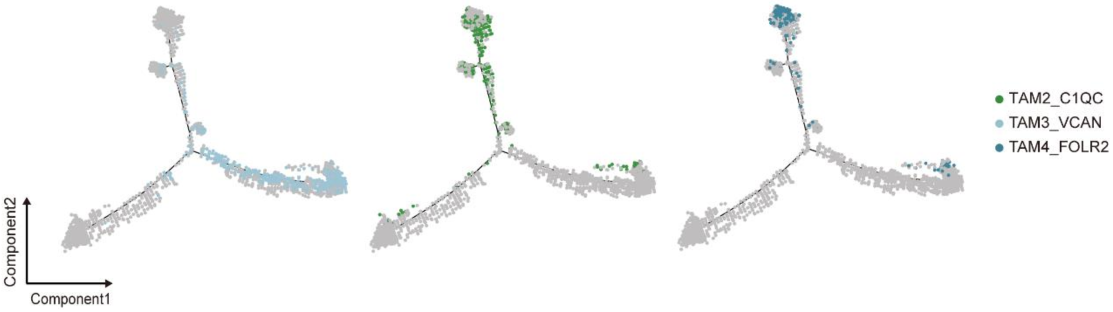

Figure S10. Plot of the trajectories labeled by subpopulations of TAMs.

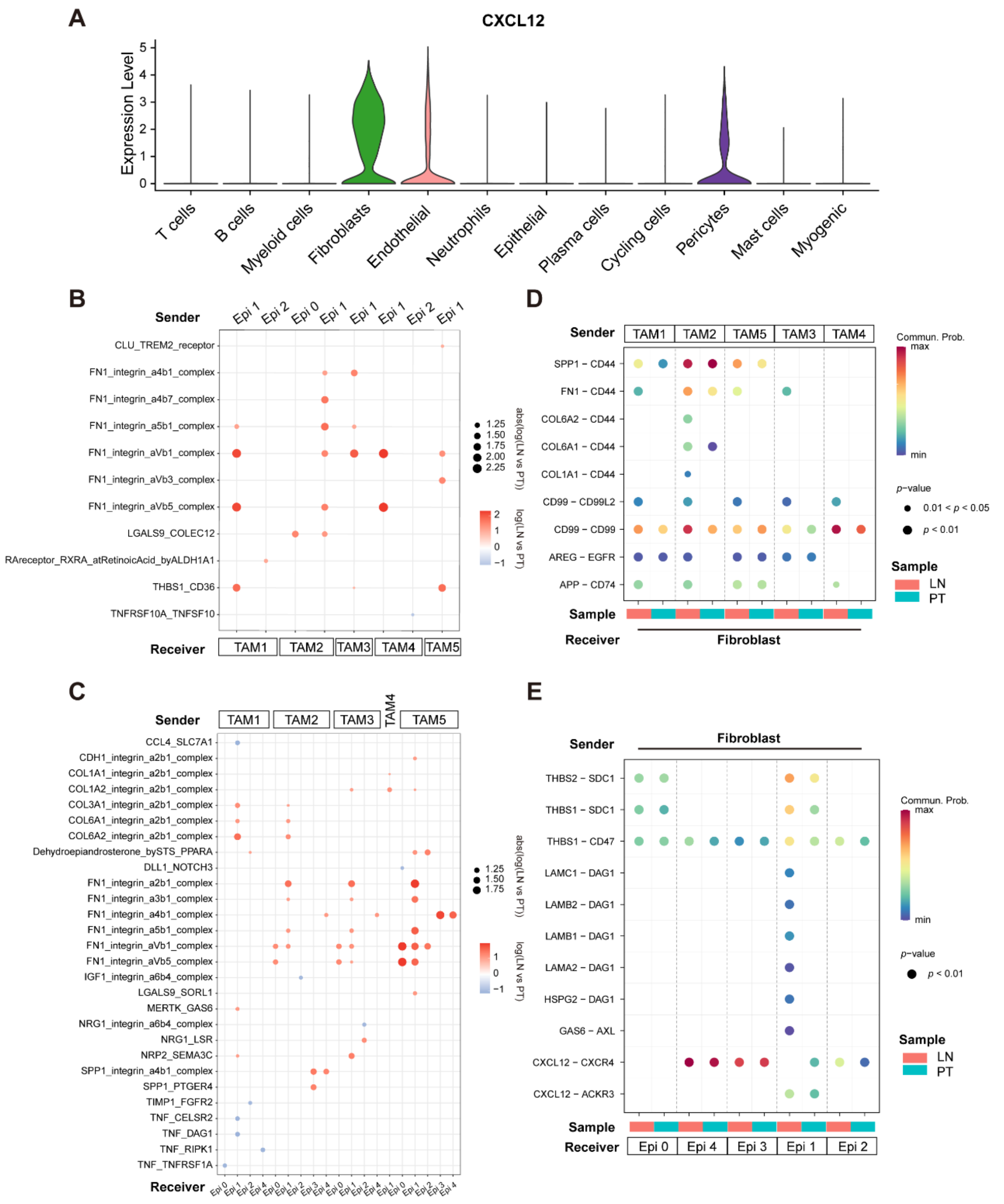

Figure S11. Interaction of TAMs and epithelial cells in LN and PT. (A) Violin plots displays the expression levels of CXCL12 in different cell types. (B) Dot plots displays selected ligand-receptor interactions in epithelial cells to TAMs. (C) Dot plots indicates selected ligand-receptor interactions in TAMs to epithelial cells. The ligand-receptor interactions and cell-cell interactions are indicates at

columns and rows, respectively. The log10(LN vs PT) were indicated by colour heatmap in one-sided permutation test. The absolute value of log10(LN vs PT) were indicated by circle size. (D) Dot plots displays selected ligand-receptor interactions in TAMs to fibroblasts. (E) Dot plots displays selected ligand-receptor interactions in fibroblast to epithelial cells.

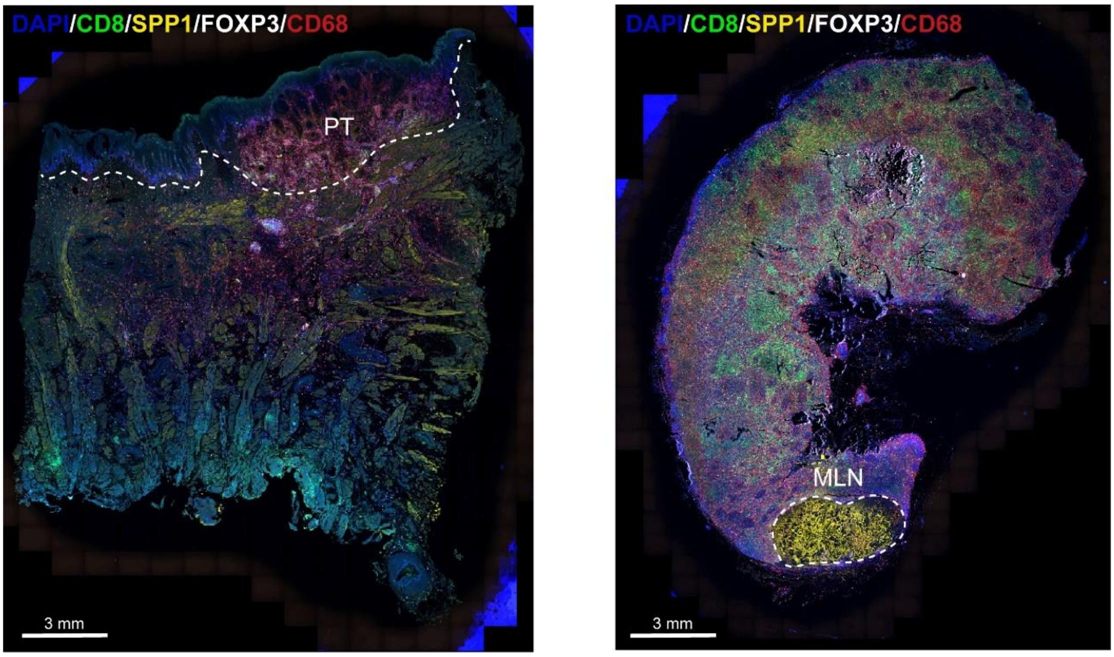

Figure S12. Whole-side scan image of a mIHC slide. A whole-side scan image of a multiplex immunohistochemical staining (mIHC) slide. Primary tumor (left), Metastasis lymph nodes (right). Scale bar: 3 mm. Dotted lines indicate the extent of primary or metastatic tumors.

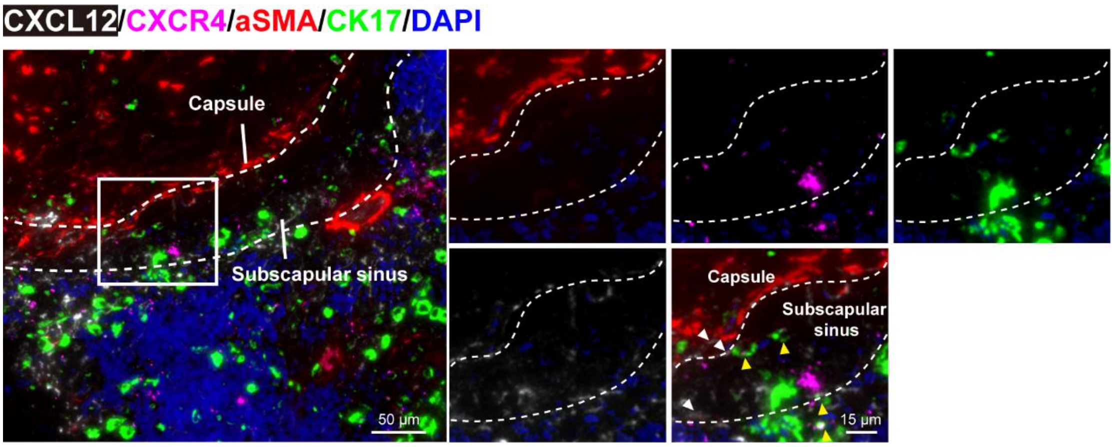

Figure S13. The spatial position of CXCR4⁺ CK17⁺ metastasized tumor cells and CXCL12⁺ aSMA⁺ fibroblasts. Dotted lines indicats the boundaries of lymph capsule and subscapular sinus respectively. White arrows indicates CXCL12⁺ aSMA⁺ fibroblasts; Yellow arrows indicates CXCR4⁺ CK17⁺ tumor cells.
